# RNA-binding protein IMP1/ZBP1 directs local translation in microglial processes to regulate motility and phagocytosis during inflammation

**DOI:** 10.1371/journal.pbio.3003463

**Published:** 2025-11-10

**Authors:** Josune Imaz-Iruretagoyena, Maite Blanco-Urrejola, Irene Núñez-García, Irene García-Toledo, Luis C. Fernández-Beltrán, Mar Márquez, Silvia Corrochano, Amanda Sierra, Jimena Baleriola

**Affiliations:** 1 Achucarro Basque Center for Neuroscience, Leioa, Spain; 2 Departamento de Neurociencias, Universidad del País Vasco (UPV/EHU), Leioa, Spain; 3 Departamento de Biología Celular e Histología, Universidad del País Vasco (UPV/EHU), Leioa, Spain; 4 Neurological Disorders Group, Hospital Clínico San Carlos, Instituto de Investigación Sanitaria Hospital Clínico San Carlos (IdiSSC), Madrid, Spain; 5 Department of Medicine, Universidad Complutense de Madrid, Madrid, Spain; 6 IKERBASQUE, Basque Foundation for Science, Bilbao, Spain; 7 Departamento de Bioquímica y Biología Molecular, Universidad del País Vasco (UPV/EHU), Leioa, Spain; 8 Cajal Neuroscience Center, Alcalá de Henares, Madrid, Spain; UCSD, UNITED STATES OF AMERICA

## Abstract

Polarized cells in the brain, such as neurons and glia, rely on the asymmetric distribution of their proteins compartmentalizing the function of dendrites, axons, glial projections, and endfeet. Subcellular proteomes can be assembled either by the transport of proteins synthesized in the cell soma or by the delivery of mRNAs to target compartments where they are locally translated into proteins. This latter mechanism is known as local protein synthesis or local translation, and it has been best studied in neurons. Increasing evidence suggests it is also required to maintain local protein homeostasis in glial cells; however, in microglia, local translation remains largely unexplored. Given the scant evidence, we aimed at exploring the existence of local translation in peripheral microglial processes (PeMPs) and unraveling its functional significance. We report that local translation indeed happens in PeMPs, and it is enhanced by triggering a microglial inflammatory response with bacterial lipopolysaccharides (LPS) suggesting a functional relevance of this molecular mechanism in response to inflammation. We found that *Actb* mRNA polarizes to PeMPs and is locally translated upon LPS exposure. Interestingly, downregulation of the *Actb*-binding protein IMP1/ZBP1 impaired *Actb* mRNA polarization and its localized translation, and led to defects in filopodia distribution, PeMP motility, lamellar directed migration, and phagocytosis in microglia. Thus, our work contributes to recent findings that mRNA localization and localized translation occur in microglia and gives a mechanistic insight into the relevance of this molecular mechanism in fundamental microglial functions in response to inflammation.

## Abstract 2

Local mRNA translation in polarized cells is well studied in neurons but remains largely unexplored in microglia. This study shows that inflammation enhances microglial RNA localization and translation via the RNA binding protein IMP1/ZBP1, regulating morphology, motility, and phagocytosis.

## Introduction

Brain cells present an extremely complex and polarized morphology with cell extensions (dendrites, axons, and glial peripheral processes) reaching more than a meter in length in the case of some human axons [1] and more than 200 µm in some rat microglial populations [2]. Such complexity is required for their specific functions, and it mirrors the equally complex structure of the organ they are hosted in. Morphological and functional polarization of brain cells is achieved by asymmetrical distribution of their proteins to distinct subcellular compartments. Subcellular proteomes are assembled either by protein transport from the soma or by local translation. The latter can be accomplished by the transport of the mRNAs to the periphery followed by their translation at the target site. This mechanism enables cells to react to environmental changes in an acute manner, as proteins are produced only when and where they are needed [3].

Although local translation is a highly conserved mechanism in eukaryotic cells [4], in the nervous system it has been mostly studied in neurons where it is involved in axon guidance, arborization, and maintenance [5], synapse formation [6], and synaptic plasticity [7]. Local translation is, however, not exclusive to neurons and increasing evidence indicates that oligodendrocytes, astrocytes, and radial glia also rely on this mechanism to maintain local protein homeostasis and their functional fitness [8]. Surprisingly, mRNA transport and localized translation in microglia have remained unexplored until only recently, when *Rpl4* mRNA, which encodes the 60S ribosomal protein L4, was detected in microglial processes in the mouse brain [9] and a localized ribosome-associated transcriptome was identified [10]. Additionally, in an *ex vivo* wound model, microglial local translation was found to be relevant for efficient phagocytosis [10]. Given the scant evidence on localized translation in microglia, this proof-of-concept article aims to contribute to shed light on the relevance of mRNA localization and local protein synthesis in regulating fundamental microglial functions in response to inflammation.

Microglia are the resident immune cells of the central nervous system, involved in brain homeostasis maintenance, and the first line of defence upon pathogen infection and brain injury. Microglia continuously survey the brain parenchyma with their peripheral processes, from hereafter PeMPs, which are continuously extending and retracting [11]. Upon pathological insults, microglia rapidly change their morphology by remodeling their actin cytoskeleton, leading to enlarged processes and even the acquisition of bushy and ameboid morphologies [12,13]. During neuroinflammation, many secreted factors induce microglial proliferation and PeMP motility toward or away from the source of injury. PeMP motility depends on two peripheral F-actin-rich filamentous structures: (1) fine processes, termed filopodia, which survey the environment and respond to the inflammatory cues or chemokines by inducing intracellular signaling, and (2) large processes, termed lamellipodia, which are responsible for process extension and/or retraction [12,14]. For example, microglia cultured in the presence of adenosine triphosphate (ATP) gradients extend their lamellae toward higher concentrations of the chemokine, while in the presence of lipopolysaccharides (LPS), processes retract moving away from the ATP source [15]. Increasing cAMP levels *ex vivo* causes filopodia to extend while inducing lamellar retraction, while *in vitro* cAMP-dependent process retraction is accompanied by microglia acquiring an ameboid phenotype [14]. Ameboid and dystrophic microglia, which are characterized by lack or fewer peripheral processes can also be found *in vivo* in rodent models and in human brains upon injury [16,17], during aging [18] and in Alzheimer’s disease [19,20]. Both lamellipodia and filopodia were already described in axonal growth cones more than three decades ago [21]. Like PeMPs, growth cone filopodia sense external cues and extend toward chemoattractants or collapse in response to molecular repellents guiding lamellae toward or away from the guidance cue. Importantly, growth cone behavior is regulated by local protein synthesis [5]. Given the similarities between axonal growth cones and PeMPs, we hypothesized that mRNA transport and local translation happen in microglia supporting essential functional changes in response to inflammation.

Here, we show that exposure of microglia to an inflammatory challenge driven by bacterial LPS induces the localization of *Actb* mRNA to PeMPs both *in vitro* and *in vivo*, as well as its localized translation in primary microglial cultures. β-actin local synthesis has been involved in cell motility in fibroblasts, and in dendritic spine rearrangements and growth cone behavior in neurons [22,23]. One of the RNA-binding proteins (RBPs) responsible for *Actb* localization is the well-characterized IGF2 mRNA-binding protein 1/zipcode-binding protein 1 (IMP1/ZBP1) [24]. Importantly, *Imp1* knockout mice show defects in *Actb*-containing granule motility in dendrites and transient *Imp1* knockdown affects the dendritic distribution of *Actb* and impairs dendritic arborization. Additionally, *Imp1* haploinsufficiency decreases *Actb* mRNA localization to axons and limits the regeneration of peripheral nerves upon injury [25–27]. Interestingly, when we downregulated IMP1/ZBP1 in microglia, *Actb* localization and β-actin synthesis was decreased in PeMPs, and resulted in impaired filopodia distribution, PeMP motility, lamellar polarized migration, and phagocytosis upon LPS exposure. Therefore, our results suggest for the first time that IMP1/ZBP1-dependent mRNA localization is relevant for the microglial response to inflammation.

## Results

### LPS induces local protein synthesis in peripheral microglial processes (PeMPs)

**Fig 1:**
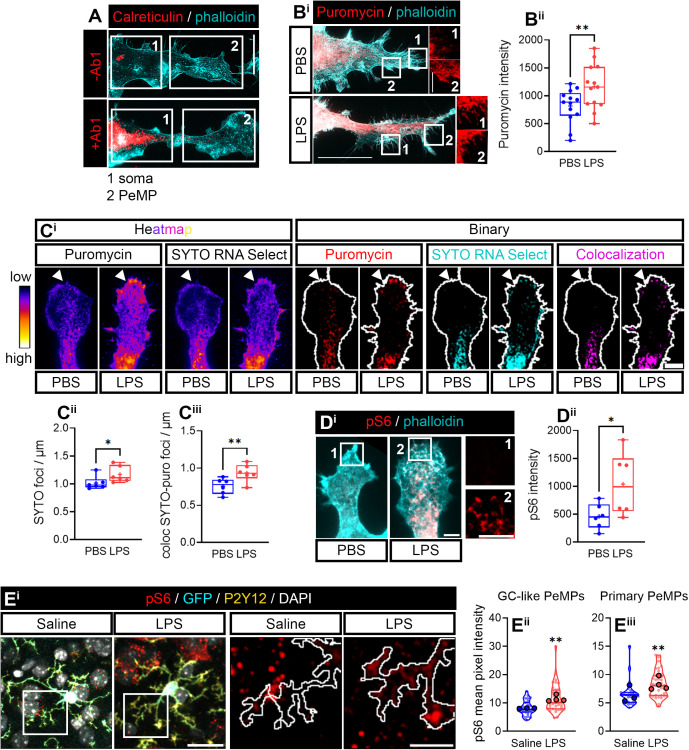
Newly synthesized proteins increase in microglial peripheral structures upon LPS exposure. **(A)** To identify the somatic domain and the peripheral structures in microglia, cells were stained with an anti-calreticulin (Calr) antibody (+Ab1) to visualize the “somatic” ER (soma, 1) and with phalloidin to visualize the actin-dense lamellipodia (2). The periphery was defined as the region where Calr was undetectable compared to a no-primary antibody negative control (−Ab1) which coincides with an intense phalloidin staining. Representative micrographs of the distribution of Calr and phalloidin are shown. Scale bar, 20 µm. **(B)** Newly synthesized proteins measured in the periphery of microglia treated with vehicle (PBS) or LPS for 24 h. Puromycilated proteins in the periphery of phalloidin-stained microglia are shown. Insets (1 and 2) show puromycin labeling at the edge of lamellae where filopodia emerge. Scale bar, 20 µm (insets, 5 µm) **(B**^**i**^**)**. The box and whisker graph indicates the mean fluorescence intensity of puromycin in the periphery of microglia in 13 independent cultures (*n* = 13) analyzed by two-tailed *t* test. **p < 0.01 **(B**^**ii**^). **(C)** Puromycin and SYTO-positive heatmaps are shown, as well puromycin and SYTO-positive foci in binarized images. Scale bar, 5 µm **(C**^**i**^**)**. Box and whisker graphs represent the average SYTO-positive foci **(C**^**ii**^**)** and SYTO-puromycin colocalization **(C**^**iii**^**)** in the periphery of PBS- and LPS-treated microglia from 6 independent experiments (*n* = 6) analyzed by two-tailed t tests. *p < 0.05; **p < 0.01. **(D)** Active ribosomal protein Rsp6 was analyzed. Insets show the levels of pS6 in PeMPs from PBS (1)- and LPS (2)-treated cells. Scale bars 5 µm **(D**^**i**^**)**. The box and whisker graph indicates the mean fluorescence intensity of pS6 in PeMPs in the periphery of microglia in 6 independent cultures (*n* = 6) analyzed by two-tailed *t* test. *p < 0.05 **(D**^**ii**^**)**. **(E)** pS6 s*t*aining in cortical microglia of fms-EGFP 1-month-old mice injected with saline or LPS. Insets show the levels of pS6 in GC-like PeMPs. Scale bars 20 µm (left panels), 10 µm (right panels) **(E**^**i**^**)**. Violin plots represent the mean intensity of pS6 in 27–36 sampled GC-like PeMPs **(E**^**ii**^**)** or primary processes **(E**^**iii**^**)** from 3 to 4 mice (*n* = 27–36; smaller dots. *N* = 3–4; bigger dots). Statistical analyses were performed by two-tailed *t* tests. **p < 0.01. The data underlying this Figure can be found in S1 Data.

To characterize RNA localization and local translation in microglia, we first identified PeMPs *in vitro*, which we identified as structures peripheral to the soma with stark accumulation of filamentous (F) actin and low or absent labeling of the endoplasmic reticulum-associated protein calreticulin (Fig 1A. A representative image of both microglia lamellae and filopodia is shown in S1A Fig). F-actin is virtually present in all cell types. Hence, we then wanted to determine if our primary cultures were really enriched in microglia with few contamination from other cell types. Although we did observe some GFAP-positive cells (S1B^i^ Fig), 90.71% (±3.63%) of all F-actin-positive cells (stained with phalloidin) expressed the microglia/macrophage protein Iba-1. Moreover, almost all Iba-1-expressing cells were also positive for the microglial marker P2Y12 (99.11 ± 0.25%) (S1B^i^–S1B^iv^ Fig). Thus, by labeling cells with phalloidin we can more than likely specifically identify PeMPs. We then addressed if newly synthesized proteins could be detected in PeMPs by puromycin labeling. Puromycin is an aminoacyl-tRNA analogue that incorporates into nascent polypeptide chains during elongation in a ribosome-catalyzed reaction [28], and specific anti-puromycin antibodies can be used to detect *de novo* protein synthesis. We exposed cultured microglia to 2 µM puromycin for 10 and 30 min. Nascent peptides were readily detected after a 10-min single exposure to puromycin both in the cell soma and in lamellar PeMPs, and signal increased with a 30-min puromycin pulse. In cells preincubated with 40 µM of the protein synthesis inhibitor anisomycin, the puromycin fluorescent intensity was reduced to levels similar of that of negative controls in PeMPs (S1C Fig) confirming the suitability of puromycilation assays to detect newly synthesized proteins in the periphery of microglia.

Microglia sense signals from their environment, including inflammatory cues, with their processes [10,11]. This likely requires changes in the local proteome for cells to rapidly adapt to changes in their surroundings. Thus, we sought to address if exogenously inducing inflammation regulated the levels of newly synthesized proteins in PeMPs. To that end, we exposed primary cultures to LPS for 24 h, an endotoxin commonly used to induce inflammatory responses in microglia [29], and treated cells with puromycin for the last 30 min. Puromycin fluorescence increased upon a 24-h LPS exposure in PeMPs (Fig 1B) suggesting that inflammation enhances the synthesis of proteins targeted to the periphery of microglia. A pre-requisite for local protein synthesis is the transport of mRNAs to peripheral processes and the presence of ribosomal components. To determine if 24-h LPS-induced increase of puromycin in PeMPs was a result of local translation, we addressed the presence of RNA in PeMPs in control and LPS-stimulated microglia using SYTO RNASelect, a fluorescent dye that selectively binds RNA [30] showing both a diffuse and punctate staining (S1D Fig). We analyzed SYTO-positive foci and SYTO-puromycin colocalization, as previously described [31], in microglia exposed to vehicle (phosphate-buffered saline [PBS]) or LPS for 24 h. LPS treatment increased SYTO foci (binarized images in Fig 1C^i^ and 1C^ii^) in PeMPs as well as SYTO-puromycin colocalization, especially at the edge of lamellipodia where fine processes (filopodia) emerge (binarized images in Fig 1Ci and 1Ciii). Additionally, we determined if the protein synthesis machinery was activated in LPS-treated cells. For this, we immunostained microglia with an antibody that recognizes the phosphorylated form of ribosomal protein Rsp6 (pS6). Rsp6 phosphorylation has been previously used as a readout for local protein synthesis in neuronal axons [32,33]. Our results indicate that pS6 levels were significantly increased in PeMPs from LPS-treated microglia compared to control cells (Fig 1D).

Local translation enables peripheral processes to acutely respond to external cues, and although it might be sustained over time, it is expected this mechanism to be regulated upon shorter exposure to stimuli. Thus, we asked if acute LPS stimulation also increased puromycin labeling and Rps6 phosphorylation. We treated microglia with PBS or LPS for 30 min and no changes in puromycin intensity were observed between experimental groups. However, we did detect a significant increase of pS6 in LPS-treated microglia compared to controls (S1E Fig). These results, together with the ones described in the previous paragraph, suggest that acute and more prolonged inflammation both enhance local translation in PeMPs.

Finally, to determine if local protein synthesis was enhanced in response to inflammation in a more physiologically relevant model, we intraperitoneally injected 1-month-old MacGreen mice (fms-EGFP) with 1 mg per kg of body mass of LPS in 0.9% NaCl for four consecutive days with 24 h between each injection and mice were sacrificed 24 h after the last injection. Mice injected with 0.9% NaCl were used as controls. The MacGreen strain expresses enhanced green fluorescent protein (EGFP, here GFP) under the promoter of colony-stimulating factor 1 receptor (*Csf1r*) and enables the visualization of the macrophage/microglia lineage in the brain [34]. External signs of systemic inflammation, such as ptosis, hunched posture and piloerection were already visible in LPS-injected animals 24 h after the first administration, and mice became lethargic 24 h after the second injection. None of the animals that received saline injections presented symptoms of inflammation (which are described in S19 Data as part of the Supporting Information files). Upon inspection of microglia in cortical layers IV and V, we observed that virtually all GFP-positive cells in the cortex expressed the microglia-specific protein P2Y12 (S2A Fig) indicating no infiltration of border macrophages. We then focused on this microglia population to quantify the levels of Rps6 phosphorylation in PeMPs *in vivo*. We observed that pS6 was significantly increased in response to LPS in PeMPs that resembled axonal growth cones (GC-like PeMPs. Fig 1E^i^ and 1E^ii^ and schematically represented in S2B Fig) as well as in primary processes that directly emerge from the cell soma (primary-PeMPs. Fig 1E^i^ and 1E^iii^ and schematically represented in S2B Fig) compared to saline-injected mice. Altogether, these data strongly suggest that LPS-induced inflammation enhances local protein synthesis in PeMPs both *in vitro* and *in vivo*.

### LPS drives *Actb* mRNA localization to PeMPs

**Fig 2:**
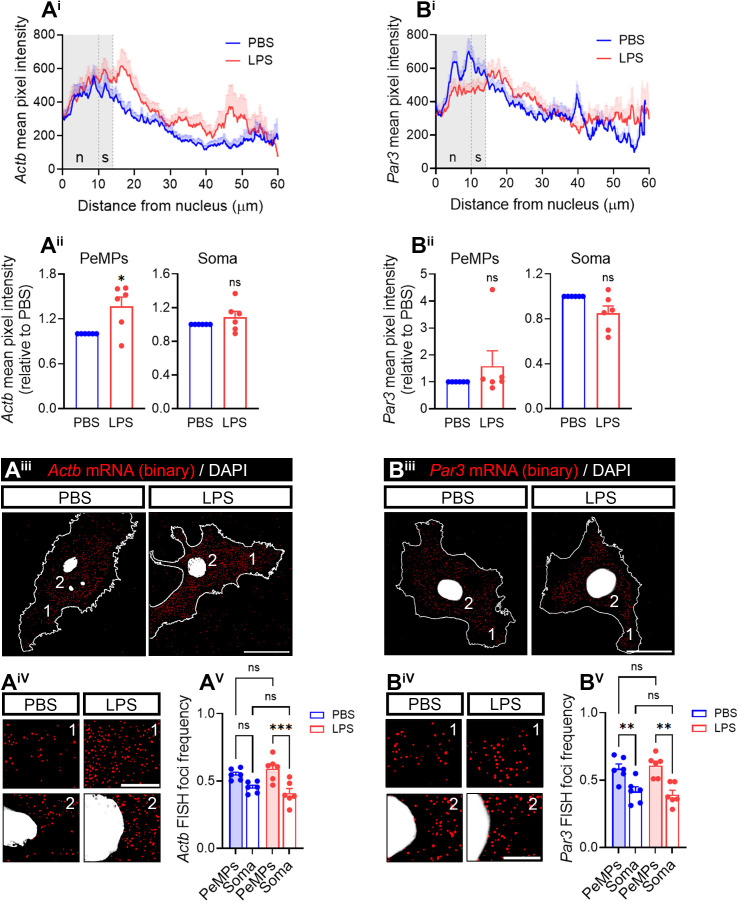
*Actb* and *Par3* mRNA in PeMPs *in vitro.* **(A)** *Actb* levels measured by FISH in primary microglia treated with PBS in LPS for 30 min. Linescans represent the mean distribution ±SEM of the FISH signal from the nucleus to the PeMPs in 45–48 individual cells per condition **(A**^**i**^**)**. Bar graphs show mean *Actb* levels ±SEM in LPS-treated cells relative to controls in PeMPs and the soma measured in 6 independent experiments (*n* = 6). Two-tailed *t* tests. *p < 0.05; n.s, not significant **(A**^**ii**^**)**. Distribution of *Actb*-positive foci in binarized images. (1) and (2) indicate the PeMPs and the perinuclear regions, respectively, represented in insets. Scale bars, 20 µm (10 µm insets) **(A**^**iii**^ **and A**^**iv**^**)**. The box and whisker graph shows the mean relative frequency distribution ±SEM of *Actb* foci in the periphery and the soma of microglia after treatment with PBS or LPS for 30 min in 6 independent experiments (*n* = 6). One-way ANOVA followed by Holm–Šídák’s multiple comparison test. ***p < 0.001; n.s, not significant **(A**^**v**^**)**. **(B)** *Par3* levels measured by FISH in primary microglia treated with PBS in LPS for 30 min. Linescans represent the mean distribution ±SEM of the FISH signal from the nucleus to the PeMPs in 49–58 individual cells per condition **(B**^**i**^**)**. Bar graphs show mean *Par3* levels ±SEM in LPS-treated cells relative to controls in PeMPs and the soma measured in 6 independent experiments (*n* = 6). Two-tailed *t* tests. n.s, not significant **(B**^**ii**^**)**. Distribution of *Par3*-positive foci is in binarized images. (1) and (2) indicate the PeMPs and the perinuclear regions, respectively, represented in insets. Scale bars, 20 µm (10 µm insets) **(B**^**iii**^ **and B**^**iv**^**)**. The box and whisker graph shows the mean relative frequency distribution ±SEM of *Par3* foci in the periphery and the soma of microglia after treatment with PBS or LPS for 30 min in 6 independent experiments (*n* = 6). One-way ANOVA followed by Holm–Šídák’s multiple comparison test. **p < 0.01; n.s, not significant **(B**^**v**^**)**. The data underlying this Figure can be found in S2 Data.

Like in the axonal growth cones, PeMPs’ fine processes (filopodia) sense external cues and extend toward chemoattractants or collapse in response to molecular repellents guiding large processes (lamellae) toward or away from the guidance cue. Importantly, growth cone behavior is regulated by local protein synthesis. In particular, local translation of *Actb* mRNA is known to be involved in axon guidance in response to external cues [35]. Given the similarities between PeMPs and axonal growth cones, we addressed to which extent this mRNA localized to PeMPs in response to LPS. We first analyzed the presence of *Actb* mRNA in PeMPs upon LPS exposure by fluorescent *in situ* hybridization (FISH). For comparative purposes, we also focused on another mRNA: *Par3*, whose local translation is required for axonal elongation in response to NGF [36]. Neither *Actb* nor *Par3* could be detected above background levels (established by the FISH signal from nontargeting probes) in PeMPs after a 24-h treatment (S3A and S3Ci^ii^ Fig). However, *Actb* mRNA was readily detected in peripheral processes in response to a 30-min exposure to LPS, while no significant detection was observed in control cells (S3B and S3C^ii^ Fig). Consistently, fluorescence from the FISH signal was significantly increased in PeMPs from LPS-treated cells compared to PBS-treated cells (Fig 2A^i^ and 2A^ii^). Additionally, frequency analysis obtained from binarized images (Fig 2A^iii^ and 2A^iv^) indicated that *Actb* granules accumulated away from the soma in LPS-treated microglia but not in controls (Fig 2A^v^). These results suggest that acute LPS exposure leads to the polarization of *Actb* mRNA toward PeMPs. On the other hand, *Par3* mRNA was detected in PeMPs in both PBS- and LPS-treated cells (S3C^ii^ Fig), and no changes in the FISH signal were observed between experimental conditions (Fig 2B^i^ and 2B^ii^), suggesting basal *Par3* localization to the periphery of microglia. Indeed, frequency analyses revealed that *Par3* foci were significantly higher in PeMPs compared to the soma both in PBS- and LPS-treated cells (Fig 2B^iii^–2B^iv^). So far, these data indicate that different transcripts distinctly respond to LPS-induced acute inflammation and that our in situ probes (at least those for *Actb* and *Par3*) are suitable to detect localized mRNAs, which are typically found at low levels in peripheral cell structures.

**Fig 3:**
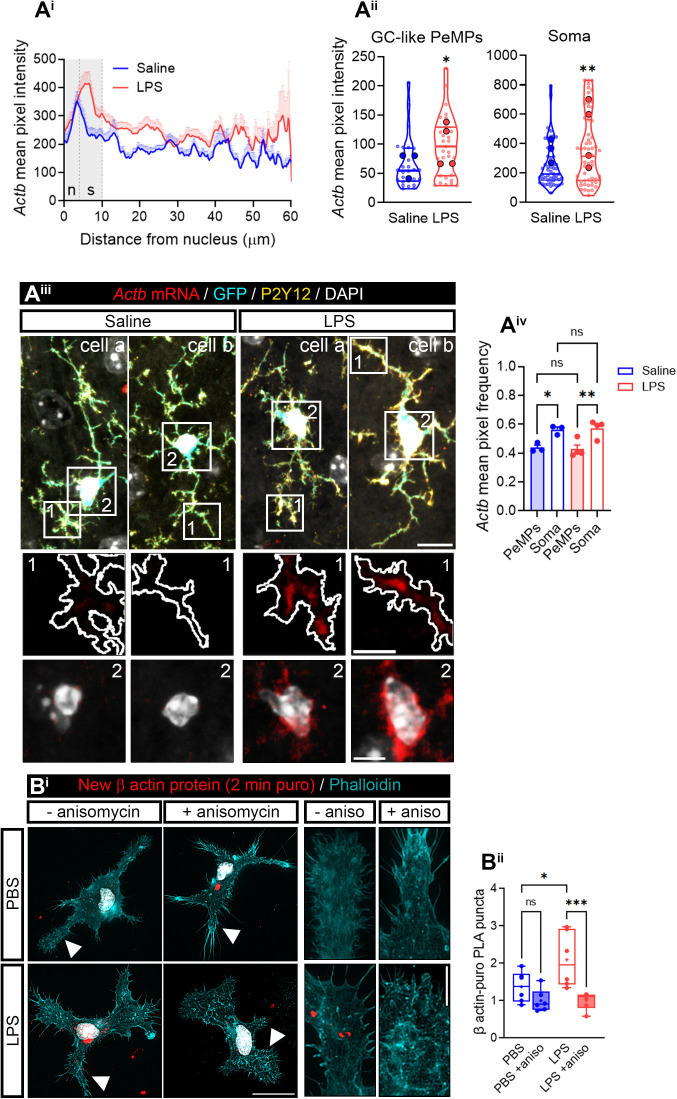
LPS increases *Actb* mRNA in PeMPs *in vivo* and enhances its localized translation *in vitro.* *Par3* mRNA in PeMPs *in vitro*. **(A)** *Actb* levels measured by FISH in cortical microglia (positive for P2Y12 and GFP) from fms-EGFP 1-month-old mice injected with saline or LPS. Linescans represent the mean distribution of the FISH signal ±SEM from the nucleus to the PeMPs in 50-58 individual cells per condition **(A**^**i**^**)**. Violin plots represent the mean intensity of *Actb* in 26–31 sampled GC-like PeMPs (smaller dots) and 50–58 cell bodies (smaller dots) from 3 to 4 mice (larger dots). Two-tailed *t* tests. *p < 0.05; **p < 0.01 **(A**^**ii**^**)**. Two cells per condition are shown as examples in fluorescence micrographs (cells a and b). (1) and (2) indicate GC-like PeMPs and cell bodies represented in insets. Scale bars 10 µm (5 µm insets) **(A**^**iii**^**)**. The bar graph shows the mean relative frequency distribution ±SEM of *Actb* intensity in PeMPs and the soma in cortical microglia from 3 to 4 mice (*N* = 3–4). One-way ANOVA followed by Holm–Šídák’s multiple comparison test. *p < 0.05; **p < 0.01 **(A**^**iv**^**)**. **(B)** New synthesis of β-actin in microglial peripheral structures was assessed with a 2-min puromycin pulse in PBS- and LPS-treated cells followed by proximity ligation assay (PLA) with antibodies against puromycin and β-actin. As a negative control, cells were preincubated with the protein synthesis inhibitor anisomycin. Micrographs show the PLA signal in microglia labeled with phalloidin. Arrowheads indicate PeMPs depicted in insets (4 rightmost panels). Scale bars 20 µm (insets 10 µm) **(B**^**i**^**)**. The box and whisker graph represents the average PLA puncta within lamellae in phalloidin-stained microglia treated with PBS or LPS in 6 independent cultures (*n* = 6). One-way ANOVA followed by Holm–Šídák’s multiple comparison test. *p < 0.05; ***p < 0.001; n.s, not significant **(B**^**ii**^**)**. The data underlying this Figure can be found in S3 Data.

We further focused on *Actb* and *Par3*, and we asked whether these transcripts were regulated by LPS *in vivo*. We performed FISH in the cortex of mice exposed to saline or LPS by IP injections and focused on the microglial population positive for both GFP and P2Y12. As shown in the linescans, we observed a generalized increase of *Actb* in all cell compartments (Fig 3A^i^) in response to LPS, including GC-like PeMPs and the soma (Fig 3A^ii^). We obtained very similar results for *Par3* mRNA (S4A–S4C Fig). However, unlike *in vitro*, *Actb* and *Par3* polarization was unclear as the intensity from the FISH signal was higher in the soma than in PeMPs, both in saline- and LPS-injected mice. Despite these discrepancies, which are likely a result on differences in the experimental approaches, we concluded that at least in the case of *Actb*, LPS enhances mRNA levels in PeMPs both *in vitro* and *in vivo* (Figs 2 and 3).

Although we used LPS and an inducer of inflammation, chemokines such as ATP are known to promote PeMPs extension and cell body migration, being both events required for the inflammatory response [37]. Thus, we addressed whether ATP also affected *Actb* polarization toward PeMPs *in vitro*. Cells were treated with 300 mM ATP for 30 min. *Actb* mRNA was neither significantly changed in peripheral processes nor in the soma of ATP-treated cells compared to control cells (S5Ai and S5A^ii^ Fig). Also, looking at the relative *Actb* distribution in binarized images (S5B^i^ Fig), the mRNA was mainly restricted to the soma in PBS-treated cells in these experiments. Similarly to previous experiments, LPS induced *Actb* polarization toward PeMPs, but this effect was unclear in response to acute ATP treatment (S5B^ii^ Fig).

We next took advantage of the presence of few Iba-1-negative cells in our cultures (likely astrocytes), (S1B^i^ Fig) to determine if they too responded to acute LPS. Pixel intensity linescans showed a minute increase in *Actb* mRNA levels in the periphery (S5C^i^ Fig), although no significant changes were observed in average (S5C^ii^ Fig). Conversely, 30-min LPS treatment did induce a significant increase in *Actb* in peripheral processes from Iba-1-positive cells (S5Ci^ii^ Fig), suggesting a specific response of microglia to LPS.

In summary, acute inflammation induces the localization of *Actb* mRNA toward PeMPs both *in vitro* and *in vivo*, and our results suggest this effect is selectively driven by LPS but not by ATP, at least at the dose tested.

### LPS induces *Actb* and *Par3* localized translation in PeMPs

To address if *Actb* polarization upon acute LPS exposure *in vitro* was accompanied by changes in local translation, we performed proximity ligation assays (PLAs) combining specific antibodies against puromycin and β-actin protein. Again, for comparison, we also addressed *Par3* local translation. PLA enables the detection of newly synthesized target proteins during the timeframe of puromycin labeling [38]. Since levels of newly produced proteins are typically lower in the processes than their soma-produced counterparts [31], to ensure the PLA signal was protein synthesis-specific, we exposed cultured microglia to PBS or LPS for 30 min and co-incubated the cells with vehicle or with 40 µM of the protein synthesis inhibitor anisomycin. Nascent polypeptides were labeled with puromycin for the last 2 or 10 min of treatment. Levels of newly synthesized β-actin (S6A and 3B Figs) and Par3 (S6B Fig) were blocked by anisomycin in LPS-treated cells but not in PBS-treated cells. Although we cannot fully rule out that both proteins appear in PeMPs by their diffusion or transport from the soma, the β-actin PLA signal following a puromycin pulse as short as 2 min is highly suggestive of LPS inducing the localized translation of *Actb* mRNA. These results also indicate that while *Actb* localization might be a limiting step for β-actin local synthesis in response to LPS, this inflammatory cue might enhance local translation of pre-existing *Par3* mRNA in PeMPs.

### RNA-binding protein IMP1/ZBP1 drives *Actb* mRNA localization and localized translation in PeMPs

Zipcode-binding protein 1 (IMP1/ZBP1) was the first RBP associated to the localization of *Actb* to fibroblast lamellipodia [24]. Since then, IMP1-dependent asymmetric distribution of *Actb* has been described in many cell types, including neurons. Given the role of IMP1/ZBP1 in *Actb* mRNA localization, we aimed to explore if it played a similar role in microglia.

**Fig 4:**
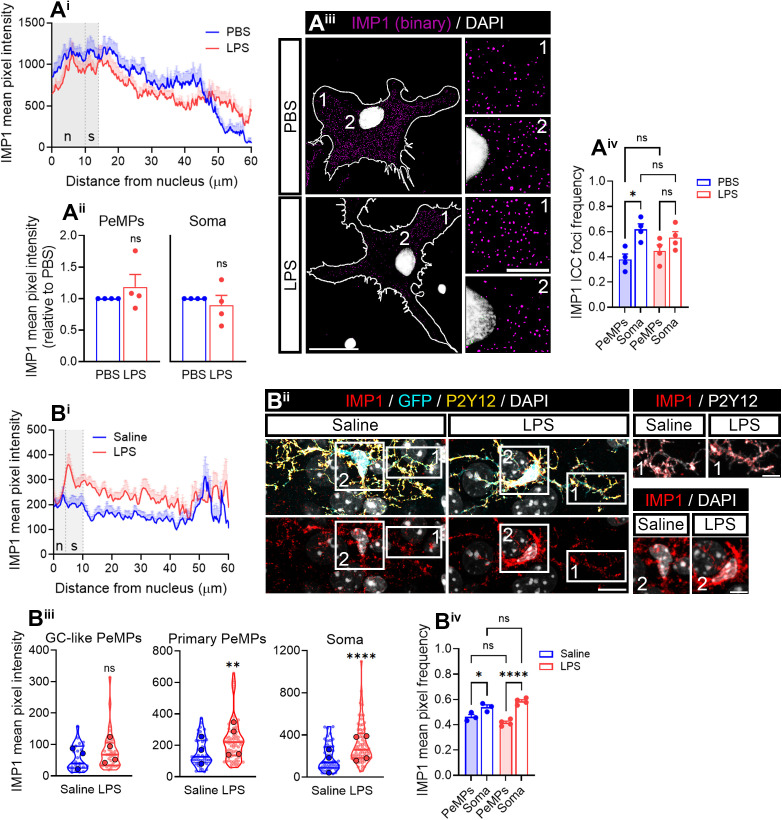
LPS regulates RNA-binding protein IMP1/ZBP1 in microglia. **(A)** IMP1/ZBP1 relative distribution in microglia. Linescans represent the mean distribution ±SEM of the IMP1 fluorescence signal from the nucleus to the PeMPs in 40 individual cells per condition **(A**^**i**^). Bar graphs show mean ±SEM IMP1/ZBP1 levels in LPS-treated cells relative to controls in PeMPs and the soma measured in 4 independent experiments (*n* = 4). Two-tailed *t* tests. n.s, not significant **(A**^**ii**^). Micrographs (binarized images) show the distribution of IMP1 in PBS- and LPS-treated cells. (1) and (2) indicate the PeMPs and the perinuclear regions, respectively, represented in insets. Scale bars, 20 µm (10 µm insets) **(A**^**iii**^). The bar graph shows the mean relative frequency distribution ±SEM of IMP1 foci in the periphery and the soma of microglia after treatment with PBS or LPS for 30 min in 4 independent experiments (*n* = 4). One-way ANOVA followed by Holm–Šídák’s multiple comparison test. *p < 0.05; n.s, not significant **(A**^**iv**^). **(B)** IMP1 levels measured by immunohistochemistry in cortical microglia (positive for P2Y12 and GFP) from fms-EGFP 1-month-old mice injected with saline or LPS. Linescans represent the mean distribution ±SEM of the fluorescent signal from the nucleus to the PeMPs in 55–72 individual cells per condition **(B**^**i**^). (1) and (2) in micrographs indicate PeMPs (both GC-like and primary processes) and cell bodies, respectively, shown in insets. Scale bars 10 µm (5 µm insets) **(A**^**ii**^). Violin plots represent the intensity of IMP1 in 28–36 sampled GC-like PeMPs (smaller dots), 55–72 primary PeMPs (smaller dots), and 55–72 cell bodies (smaller dots) from 3 to 4 mice (larger dots). Two-tailed *t* tests. **p < 0.01; ****p < 0.0001; n.s, not significant **(B**^**iii**^). The bar graph shows the mean relative frequency distribution ±SEM of IMP1/ZBP1 intensity in PeMPs and the soma in cortical microglia from 3 to 4 mice (*N *= 3–4). One-way ANOVA followed by Holm–Šídák’s multiple comparison test. *p < 0.05; ****p < 0.0001; n.s, not significant **(B**^**iv**^). The data underlying this Figure can be found in S4 Data.

First, we analyzed the levels of IMP1/ZBP1 in microglia and observed a granular pattern in the cytoplasm (S6C^i^ Fig). A nonsignificant trend toward a decrease was found upon LPS exposure (S6C^ii^ Fig). These results were further confirmed by western blotting (S6D^i^ Fig and S6D^ii^). Although no changes were observed in PeMPs (Fig 4A^i^ and 4A^ii^), IMP1/ZBP1-positive foci were restricted to the somatic region in control cells whereas upon LPS treatment they distributed more peripherally (Fig 4Aiii and 4A^iv^), partially mirroring the change in *Actb* distribution upon LPS exposure in vitro (Fig 2A). Importantly, IMP1/ZBP1 was generally increased in microglia *in vivo* in LPS-injected mice (Fig 4B^i^ and 4B^ii^), and despite these changes were not observed in GC-like PeMPs they did affect primary processes and the soma (Fig 4B^iii^). Levels of this RBP were higher in the soma than in PeMPs in both saline- and LPS-injected animals (Fig 4B^iv^). This results again partially reproduced *Actb* mRNA pattern in response to systemic inflammation *in vivo* (Fig 2C).

**Fig 5:**
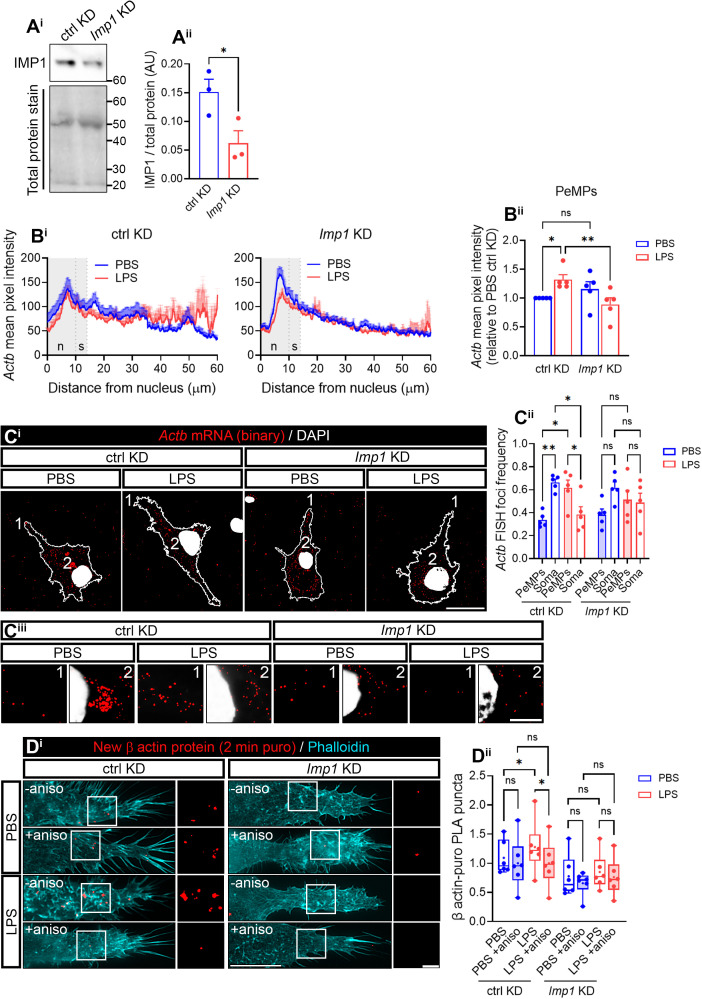
IMP1/ZBP1 regulates *Actb* mRNA localization and localized translation in microglia. **(A)** IMP1/ZBP1 downregulation upon microglia transfection with an *Imp1*-targeting siRNA. Representative western blot showing IMP1/ZBP1 and total protein levels following transfection with a control (ctrl KD) and an *Imp1*-targeting siRNA (*Imp1* KD) **(A**^**i**^**)**. The bar graph summarizes the quantification of western blots obtained from 3 independent cultures (*n* = 3). Two-tailed *t* test; n.s, not significant **(A**^**ii**^**)**. **(B)** *Imp1* knockdown (KD) alters *Actb* mRNA localization toward the periphery of microglia. Linescans show the mean distribution ±SEM of the *Actb* FISH signal from the nucleus to the PeMPs in 38–48 individual cells per condition **(B**^**i**^**)**. The bar graph represents the relative *Actb* intensity (compared to control-transfected cells exposed to PBS) in PeMPs from microglia treated with PBS or LPS and transfected with a control (ctrl KD) or an *Imp1*-targeting *(Imp1* KD) siRNA in 5 independent experiments (*n* = 5). One-way ANOVA followed by Holm–Šídák’s multiple comparison test. *p < 0.05; **p < 0.01; n.s, not significant **(B**^**ii**^**)**. **(C)** Relative distribution of *Actb* foci in binarized images. (1) and (2) indicate the PeMPs and the perinuclear regions, respectively, **(C**^**i**^**)** represented in insets **(C**^**ii**^**)**. Scale bars, 20 µm (C^i^) and 10 µm (C^ii^). The relative frequency distribution of *Actb* foci in the periphery and the soma of microglia transfected with a control (ctrl KD) siRNA or an *Imp1* siRNA (*Imp1* KD) and exposed to PBS or LPS for 30 min in 5 independent experiments (*n* = 5) is plotted in **(C**^**iii**^**)**. Two-way ANOVA followed by Holm–Šídák’s multiple comparison test. *p < 0.05; **p < 0.01; n.s, not significant. **(D)** *Imp1* KD blocks LPS-induced localized translation in microglia. Local β-actin synthesis was assessed with a 2-min puromycin pulse in PBS- and LPS-treated cells and transfected with control (ctrl KD) or *Imp1* siRNAs (*Imp1* KD), followed by PLA with antibodies against puromycin and β-actin. As a negative control, cells were preincubated with the protein synthesis inhibitor anisomycin. Representative images of microglial PLA-labeled lamellae and stained with phalloidin are shown. Scale bar, 10 µm (insets, 5µm) **(D**^**i**^**)**. The box and whisker graph represents the average of PLA puncta within lamellae in phalloidin-stained microglia cultured in 6 independent experiments (*n* = 6) analyzed by two-way ANOVA followed by Holm–Šídák’s multiple comparison test. *p < 0.05; n.s, not significant **(D**^**ii**^**)**. The data underlying this Figure can be found in S5 Data.

Since limiting the amount of IMP1/ZBP1 is sufficient to decrease *Actb* mRNA availability in axons [26], we sought to down-regulate this protein by genetic silencing in microglia. We transfected cultured microglia with two nonoverlapping small-interference RNAs (siRNAs) against *Imp1* (siRNA #1 and siRNA #2) and used a nontargeting siRNA as a negative control. Whereas siRNA #1-transfected cells showed a trend toward a decrease in IMP1/ZBP1 levels after 24-h transfection, siRNA #2 significantly downregulated the target protein both at 6 and 24 h (S6E Fig). Thus, we further used siRNA #2, hereafter *Imp1* KD, whose knockdown efficiency was verified by western blotting (Fig 5A). *Actb* mRNA was significantly increased in PeMPs in response to LPS in control-transfected cells (ctrl KD, Fig 5Bi, left linescan, and 5Bii), in line with results obtained from untransfected cells (Fig 2A). Interestingly, the effect of LPS was blocked by *Imp1* KD (Fig 5Bi, right linescan, and 5Bii). Indeed, similar results were observed when normalizing mRNA foci to the signal of a negative probe (S6F Fig). Furthermore, *Actb* mRNA foci frequency was lower in the soma of LPS- compared to PBS-treated microglia and higher in the periphery in ctrl KD cells. *Imp1* KD led to a loss of *Actb* polarization toward PeMPs in LPS-treated microglia (Fig 5Ci–5Ciii).

Next, we sought to determine if *Imp1* KD also affected local β-actin synthesis in PeMPs. We performed PLA assays to detect newly synthesized β-actin following a 2-min puromycin pulse. Like in untransfected cells (S6A and 3B Figs), acute LPS exposure in control-transfected cells led to an increase in local *Actb* translation, which was blocked by preincubation with 40 µM anisomycin. *Imp1* KD overall decreased β-actin PLA signal levels (two-way ANOVA *p* < 0.05) and no detectable protein synthesis was observed neither in PBS-treated cells nor in LPS-treated cells above levels observed in the presence of anisomycin (Fig 5D).

Altogether, these results suggest that IMP1/ZBP1 regulates *Actb* mRNA localization to PeMPs and its localized translation in response to LPS.

### *Imp1* knockdown impairs mRNAs in PeMPs which encode proteins involved in cytoskeleton regulation

**Fig 6:**
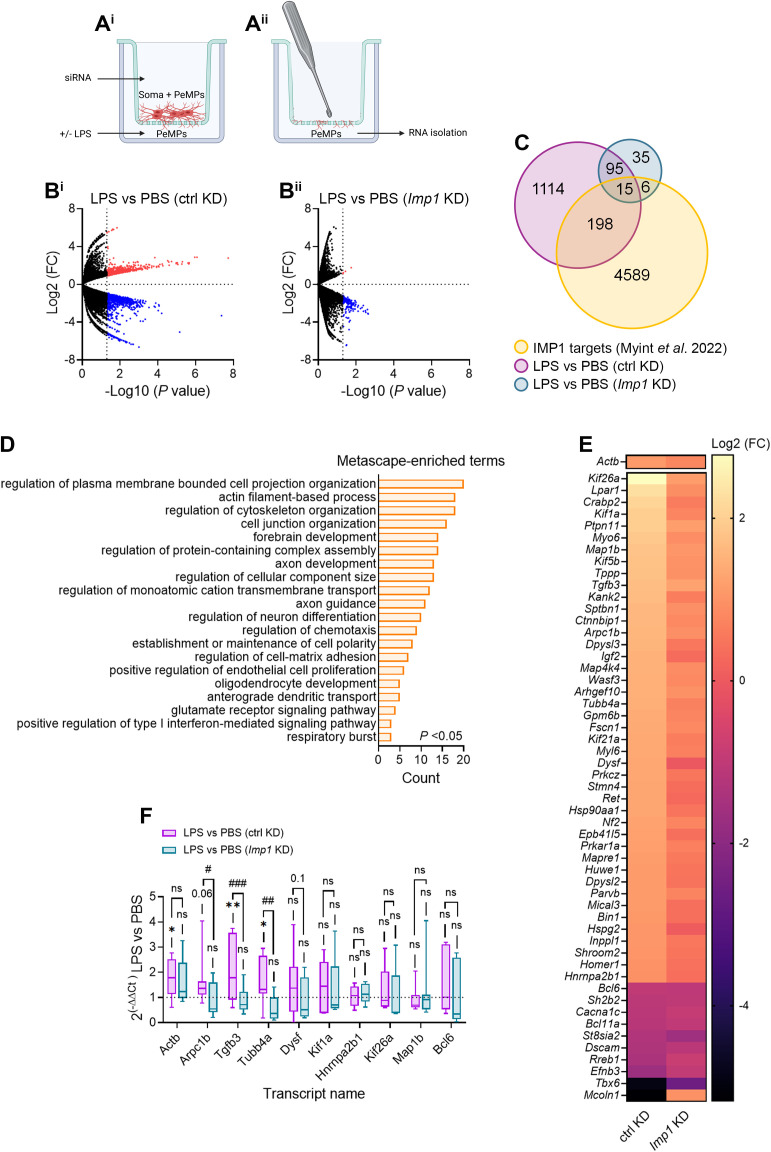
IMP1/ZBP1 inhibition affects transcripts involved in motility and cytoskeleton regulation. **(A)** Schematic representation of a transwell with 1-µm-diameter pore used for the isolation of PeMPs **(A**^**i**^**)**. Cell material from the upper compartment was removed with a cotton swab and total RNA was isolated from PeMPs (lower panel) **(A**^**ii**^**)**. Figure was created in BioRender. Baleriola, J. (2025) https://BioRender.com/urvkdx5. **(B)** Significantly regulated RNAs in response to LPS in ctrl KD **(B**^**i**^**)** and *Imp1* KD cells **(B**^**ii**^**)**. Volcano plots represent the upregulated (red dots) and downregulated (blue dots) transcripts obtained from 2 independent experiments. **(C)** Venn diagram showing the overlap between differentially localized transcripts upon LPS treatment in ctrl KD (magenta) or *Imp1* KD (cyan) microglia and previously identified IMP1 targets (yellow). **(D)** Functional annotation with Metascape of the 198 transcripts whose regulation is lost by *Imp1* knockdown. Only the top 20 categories from all significantly changed terms (*p* < 0.05) are represented in the figure. **(E)** Heatmaps showing the relative levels (LPS vs. PBS) of transcripts belonging to the 3 most enriched categories in (C) in ctrl KD and *Imp1* KD microglia. **(F)** Relative levels (LPS vs. PBS) of selected transcripts measured in 4–7 biological replicates by RT-qPCR. *p < 0.05; **p < 0.01; n.s, not significant comparing LPS- vs. PBS-treated cells transfected with control or *Imp1* siRNA. ^##^*p* < 0.01; ^###^*p* < 0.001; n.s, not significant comparing control vs. *Imp1* siRNA in LPS-treated cells. The data underlying this Figure can be found in S6 Data.

One single RBP can bind hundreds of mRNAs. Thus, we next asked whether *Imp1* KD affected other transcripts besides *Actb* either directly or indirectly. To this end, we performed RNA-Seq analyses in PeMPs isolated in a transwell system which consists of a culture insert with a polyethylene terephthalate membrane that creates two compartments. Cells were seeded on top of a 1 µm-pore membrane (upper compartment), which enables microglial PeMP extension toward the lower compartment restricting cell body migration. Microglia were transfected with a control siRNA or an *Imp1*-targeting siRNA, and the lower compartment was then treated with vehicle or LPS for 30 min (Fig 6A^i^). After removing the cell material from the upper compartment, we isolated total RNA from PeMPs (Fig 6A^ii^). RNA libraries containing both coding (mRNAs) and noncoding RNAs were prepared. Sequence alignment was conducted with HISAT2 [39], and we first analyzed the data for *Actb* itself. Although no significant differences were detected in LPS-treated cells compared to PBS treatment in ctrl KD cells, a trend toward a 1.7-fold increase was observed (*p* = 0.13). Similar fold-changes were measured by *in situ* hybridization in microglia that better responded to LPS treatment (Figs 2A^ii^ and 5A^ii^). *Imp1* KD narrowed the differences between LPS- and PBS-treated cells (1.2-fold increase, *p* = 0.68), again in accordance with our FISH experiments (Fig 5A^ii^). We then focused on differentially localized transcripts based on DESeq2 [40] analyses. PeMPs from control-transfected microglia showed significant differences in 1,596 transcripts, of which 824 were increased and 772 were decreased in response to LPS (Fig 6B^i^). Interestingly, the number of significantly regulated RNAs was greatly reduced to 174 (4 of them were increased while 170 were decreased) upon *Imp1* knockdown (Fig 6B^ii^). These results already suggest that LPS-induced changes in the local transcriptome heavily rely on IMP1. We then clustered the identified transcripts into gene ontology (GO) terms using Metascape [41]. Most regulated transcripts were involved in the immune response, both in ctrl KD and *Imp1* KD cells (S7A^i^ and S7A^ii^ Fig). Interestingly, however, while in the former LPS also induced changes in cell projection organization, locomotion, or phagocytosis, these GO terms were no longer represented in *Imp1* KD cells (S7A^i^ and S7A^ii^ Fig)

We then compared the significantly regulated RNAs in our datasets to previously published IMP1 targets [42]. We found an overlap of 213 transcripts in control-transfected cells, whereas *Imp1* knockdown led to a loss of LPS-induced regulation of more than 90% of IMP1 targets (Fig 6C). Among these 90%, we found transcripts involved in cytoskeleton regulation (3 most enriched categories in the Metascape analysis in Fig 6D). From these, ca. 81% (43 out of 53) were upregulated in response to LPS and the remaining 19% were reduced. IMP1 downregulation led to a generalized blockade of LPS-induced changes in cytoskeleton-related transcripts. Finally, we asked which of these RNAs functionally interacted with β-actin. We clustered *Actb* itself with the 53 transcripts encoding cytoskeletal proteins with STRING [43]. Functional analysis identified 28 *Actb*-interactors among the transcripts whose LPS-induced regulation was blocked in *Imp1* KD cells (S7B Fig). Reverse-transcription quantitative polymerase chain reaction (RT-qPCR) analyses were performed for *Actb* and 9 of its interactors. We confirmed that LPS-induced *Actb* localization in PeMPs was blocked by IMP1 downregulation, in line with our FISH analyses (Fig 5A^ii^). Additionally, *Arpc1b*, *Tgfb3*, and *Tubb4a* were decreased upon IMP1 downregulation in LPS-treated cells and a trend toward a downregulation was also observed for *Dysf* (*p* = 0.1. Fig 6F).

These results indicate that LPS induces the localization to PeMPs of *Actb* and other functionally related transcripts involved in cytoskeleton regulation, in an IMP1-dependent manner.

### LPS induces morphological changes in microglia and enhances random motility and polarized PeMP extension in an IMP1/ZBP1-dependent manner

**Fig 7:**
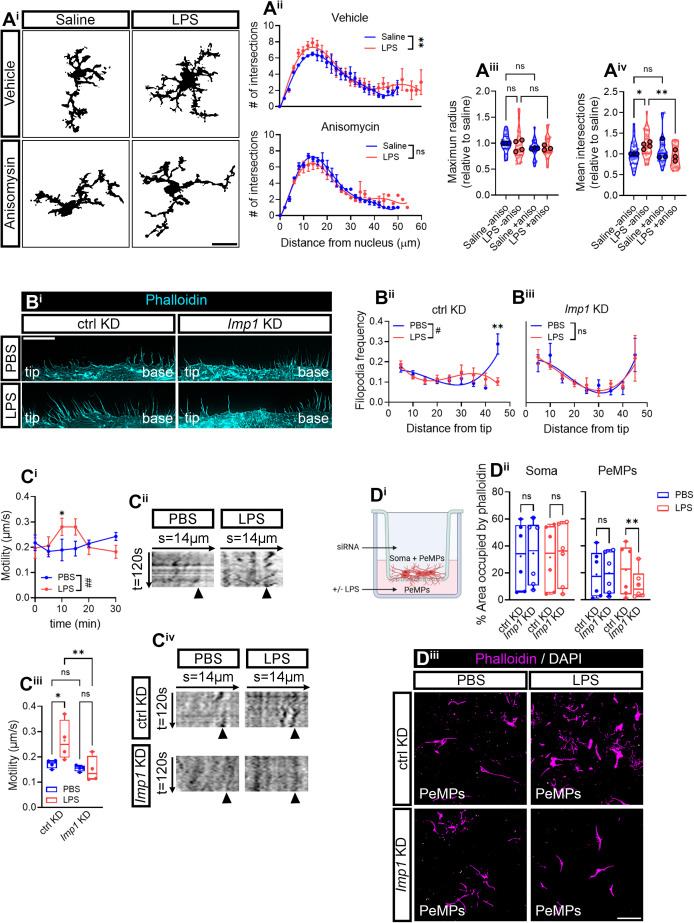
LPS-induced PeMPs morphological changes depend on IMP1/ZBP1 levels. **(A)** 2D Sholl analyses of cortical microglia from fms-EGFP 1-month-old mice injected with saline or LPS and co-injected with vehicle or anisomycin. Micrographs exemplify binarized cells used for Sholl analyses. Scale bar 20 µm **(A**^**i**^**)**. Graphs represent Sholl analyses performed in 3–4 animals per experimental group (*N* = 3–4). For simplification purposes, animals injected with vehicle (upper graph) or anisomycin (lower graph) are represented separately, although statistical analysis using two-way ANOVA with Holm–Šídák’s *post hoc* test was used taking all experimental groups into account. **p < 0.01; n.s, not significant **(A**^**ii**^**)**. PeMP extension in response to LPS *in vivo* was measured as the cell maximum radius of 25–36 cortical microglia (smaller dots) from 3 to 4 animals (larger dots). One-way ANOVA followed by Holm–Šídák’s multiple comparison test. n.s, not significant **(A**^**iii**^**)**. Mean intersections from 25 to 36 cortical microglia (smaller dots) measured in 3–4 animals (larger dots) are represented in violin plots in **(A**^**iv**^**)**. One-way ANOVA followed by Holm–Šídák’s multiple comparison test. *p < 0.05; **p < 0.01; n.s, not significant. **(B)** Distribution of filopodia along microglial lamellae in control (ctrl KD) and *Imp1* siRNA-transfected cells (*Imp1* KD) exposed to PBS or LPS for 30 min. Representative micrographs depicting filopodia distribution in phalloidin-stained microglia are shown. Scale bar, 10 µm **(B**^**i**^**)**. Graphs represent the relative filopodia distribution of microglia cultured in 6 independent experiments (*n* = 6). Two-way ANOVA followed by Holm–Šídák’s multiple comparison test. #*p* < 0.05 (factor interaction); *p < 0.05; n.s, not significant **(B**^**ii**^**)**. **(C)** Random PeMP motility measured in response to LPS in untransfected- and siRNA-transfected cells. Live cell imaging was performed in microglia exposed to PBS or LPS for different times and PeMP motility was assessed for 2 min every 5 s. Time-course graphs represent PeMP motility in response to PBS or LPS in 4–6 independent cultures from untransfected cells (*n* = 4–6). Two-way ANOVA followed by Holm–Šídák’s multiple comparison test. #*p* < 0.05 (factor interaction); *p < 0.05; n.s, not significant **(C**^**i**^**)**. Kymographs show the representative oscillations of microglial lamellae during 2 min imaging (5 s cycles) after a 10-min treatment with vehicle or LPS **(C**^**ii**^**)**. Box and whisker graph show the mean PeMP motility in control-transfected (ctrl KD) and *Imp1* KD cells exposed to vehicle or LPS for 10 min from 4 independent cultures (*n* = 4). One-way ANOVA with Holm–Šídák’s *post hoc* test **(C**^**iii**^**)**. Kymographs show the representative oscillations of microglial lamellae during 2 min imaging (5 s cycles) after a 10-min treatment with PBS or LPS in control- or IMP1-siRNA-transfected microglia **(C**^**iv**^**)**. **(D)** To address lamellar migration, we restricted the soma to the upper compartment by culturing cells in 1-µm-diameter transwell membranes. PBS or LPS were applied to the bottom to the lower compartment for 30 min. Figure was created with BioRender.com **(D**^**i**^**)**. The box and whisker graphs represent the relative coverage of phalloidin staining at the upper (Soma; left) and the lower (PeMPs; right) sides of the membrane. Analyses were performed in 6 independent experiments (*n* = 6) by one-way ANOVA followed by Holm–Šídák’s multiple comparison test. **p < 0.01; n.s, not significant **(D**^**ii**^**)**. Representative images of lamellae migrated to the lower side of the membrane are shown. Scale bar, 50 µm **(D**^**iii**^**)**. The data underlying this Figure can be found in S7 Data.

*Actb* mRNA is the prototypical example of a localized transcript in many eukaryotic cells, and its local translation is involved in cytoskeletal rearrangements, cell polarity, and lamellar migration toward attractant cues [44]. In our system, IMP1/ZBP1 downregulation alters *Actb* mRNA localization to PeMPs and its localized translation (Fig 5), and it impairs other actin cytoskeleton regulators (Fig 6) *in vitro*. Thus, our next step was to address any morphological changes in microglia occurring in response to LPS treatment and when local *Actb* availability was limited in microglial processes by *Imp1* knockdown. To that end, we first performed morphological analyses in cortical microglia from fms-EGFP mice injected with saline or LPS. Upon LPS administration, microglia in cortical layers IV and V acquired a bushier morphology with fine processes emerging from primary processes close to the soma (Fig 7A^i^, upper panels), in line with previous observations [14], and no dystrophic or ameboid microglia were observed (S2A Fig). These results are consistent with a higher morphological complexity as quantified by 2D Sholl analyses (Fig 7A^ii^, upper graph) and with the increase in mean intersections observed in LPS-injected mice compared to saline-injected mice (Fig 7A^iv^). However, we noticed no changes in PeMP extension measured as the maximum cell radius (Fig 7A^iii^). Interestingly, morphological changes driven by LPS were no longer observed in mice that also received anisomycin (Fig 7A^i^, lower panels; Fig 7A^ii^, lower graph), and mean intersections were significantly decreased in LPS-injected animals administered with this drug (Fig 7A^iv^). These results suggest that LPS-induced morphological complexity requires protein synthesis.

To determine whether IMP1/ZBP1-dependent mRNA localization was responsible for microglial morphological changes in response inflammation, we transfected primary cultures with a control or with an *Imp1*-targeting siRNA. LPS did not alter PeMP length or width neither in untransfected cells nor in transfected microglia (S8A and S8B Fig); however, it did modify filopodia distribution along the lamellar surface. We observed that most of the fine processes were localized at the base of lamellae in PBS-treated microglia and redistributed more homogenously upon LPS exposure when cells were transfected with a control siRNA for 24 h. Conversely, filopodia distribution was similar in both experimental conditions in *Imp1*-transfected cells. These results suggest that LPS leads to the rapid rearrangement of filopodia in an IMP1/ZBP1-dependent manner (Fig 7B).

We then analyzed PeMP motility by life cell imaging in cells treated with vehicle or LPS for 0, 5, 10, 15, 20, and 30 min. Cell visualization was performed for 2 min (5-s cycles) at each time point. LPS enhanced PeMP motility after a 10-min treatment (Fig 7C^i^ and 7C^ii^), however, no net protrusion was observed (S8C Fig). To determine if IMP1/ZBP1 is required for LPS-induced motility, we transfected cells with siRNAs and, again, performed live imaging. In line with results from untransfected cells, LPS also increased random (undirected) PeMP motility in control-transfected cells, and this effect was blocked by *Imp1* KD (Figs 7C^iii^, 7C^iv^, and S8D).

All experiments reported in this section thus far were performed either by systemic injection *in vivo* or bath application of vehicle or LPS to cells *in vitro*. In this context, PeMP motility in unlikely directed as treatments are not focal, which could explain by we were not able to observe PeMP extension. Thus, as a next step, we attempted to focalize the treatments by culturing cells in transwells. Cells were seeded on top of a 1 µm-pore membrane and vehicle or LPS were applied to the lower compartment (Fig 7D^i^). In this case, LPS did enhance PeMP polarized migration measured as the area covered by F-actin in the lower compartment (S9A^i^, right graph, and S9A^ii^ Fig), whereas the upper compartment remained unaffected (Soma in S9A^i^, and S9A^ii^ Fig). Once we observed these results were reproduced in control-transfected cells (S9Ai^ii^ Fig), we analyzed the effect of *Imp1* knockdown on PeMP polarization. PeMP extension toward the lower compartment was significantly reduced in *Imp1* KD compared to ctrl KD cells exposed to LPS (PeMPs in Fig 7D^ii^ and 7D^iii^), and F-actin coverage remained unchanged in the upper compartment (Soma in Fig 7D^ii^).

Although *in vivo* cell body movement is restricted in adult microglia, and inflammatory cues lead to the retraction or extension of PeMPs, some reports *in vitro* have suggested that cultured microglia cell lines can undergo whole cell migration both basally and in response to LPS [45,46]. Thus, we wondered if LPS induced cell migration in transwells. If cell bodies were allowed to move toward the lower compartment by culturing microglia on 3 µm-pore membranes, neither LPS nor IMP1/ZBP1 downregulation affected cell migration (S9C Fig), suggesting that PeMP-directed extension and cell body migration might be regulated by different mechanisms, being the latter LPS- and IMP1-independent.

Lastly, we aimed to determine if PeMP polarized extension could also be triggered by ATP; however, we did not observe any effect with this chemokine (S9D Fig). Thus, LPS but not ATP enhances polarized PeMP motility in an IMP1/ZBP1-dependent manner.

### IMP1/ZBP1 and *Actb* mRNA are enriched in phagocytic pouches, and IMP1 downregulation impairs phagocytosed particle degradation

Phagocytosis is one of the main functions of microglia to restore brain homeostasis by removing cell debris and damaged cells upon inflammation, and it is driven by PeMPs *in vivo*. During phagocytosis, PeMPs extend toward damaged cells, rearrange their cytoskeleton, and form specialized peripheral structures termed phagocytic pouches [29]. Interestingly, Vasek and colleagues have recently described the localized translatome of microglia, and have reported that local protein synthesis is required for efficient phagocytosis [10]. Given that local β-actin production is required for rapid cytoskeletal remodeling in response to external guidance cues [35], and that our own data indicate that *Imp1* KD decreases the levels of transcripts involved in actin cytoskeleton regulation in PeMPs (including *Actb* itself, Figs 5, 6, and S7), we sought to analyze the relevance of IMP1/ZBP1 on phagocytosis.

**Fig 8:**
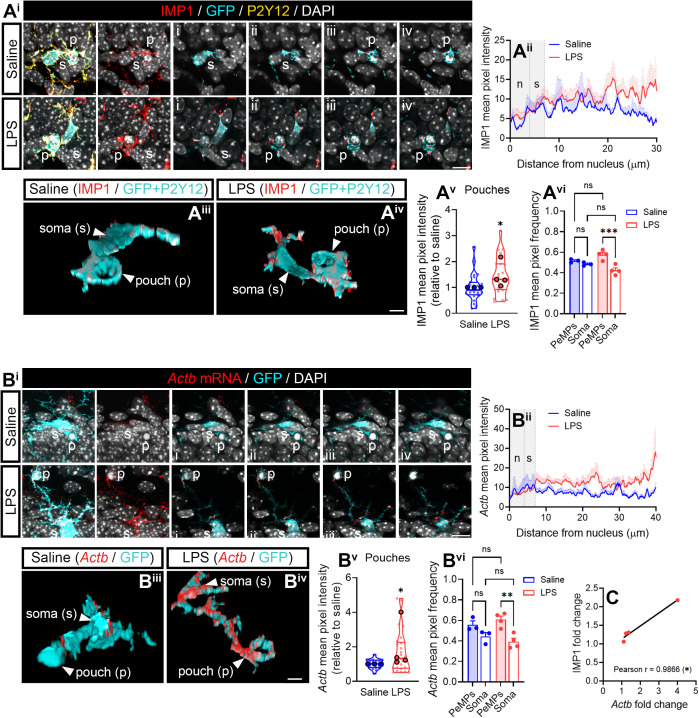
LPS induces the enrichment of IMP1 RBP and *Actb* mRNA in phagocytic pouches *in vivo.* **(A)** IMP1 levels in phagocytic microglia in the dentate gyrus. Representative micrographs of IMP1/ZBP1 immunostaining in the cell body (s) and in phagocytic PeMPs (p) of microglia from saline- and LPS-injected mice. A series of 5 consecutive optical sections (i–v) are shown. Scale bar 10 µm **(A**^**i**^**)**. Linescans represent mean ±SEM IMP1 fluorescent signal from the nucleus to phagocytic pouches in 8–12 individual cells per condition **(B**^**ii**^**)**. 3D reconstructions of microglial cells shown in **(A**^**i**^**)** and stained for IMP1 in saline- **(A**^**iii**^**)** and LPS-injected **(A**^**iv**^**)** mice. Images might have been rotated to position the phagocytic pouch in foreground. Scale bar 5 µm. Violin plots summarize the relative changes in IMP1/ZBP1 levels (LPS-injections compared to controls) observed in phagocytic PeMPs from 23 to 24 cells (smaller dots) measured in the hippocampus of 3–4 mice (larger dots). Two-tailed *t* test; *p < 0.05 **(A**^**v**^**)**. The bar graph shows the relative frequency distribution of IMP1/ZBP1 intensity in pouches and the soma of microglia from 3 to 4 mice (*N* = 3–4). One-way ANOVA followed by Holm–Šídák’s multiple comparison test. ***p > 0.001; n.s, not significant **(A**^**vi**^**)**. **(B)** *Actb* mRNA levels in phagocytic microglia in the dentate gyrus. Representative micrographs of *Actb* FISH signal in the cell body (s) and in phagocytic PeMPs (p) of microglia from saline- and LPS-injected mice are shown. Five consecutive optical sections are represented (i–v). Scale bar 10 µm **(B**^**i**^**)**. Linescans depict the mean *Actb* FISH fluorescent signal ±SEM from the nucleus to phagocytic pouches in 12–18 individual cells per condition **(B**^**ii**^**)**. 3D reconstructions of microglial cells shown in **(A**^**i**^**)** and stained with *Actb* probes in saline- **(A**^**iii**^**)** and LPS-injected **(A**^**iv**^**)** mice. Images might have been rotated to position the phagocytic pouch in foreground. Scale bar 5 µm. Violin plots summarize the relative changes in *Actb* mRNA levels (LPS-injections compared to controls) observed in phagocytic PeMPs from 16 to 25 cells (smaller dots) measured in the hippocampus of 3–4 mice (larger dots). Two-tailed *t* test; *p < 0.05 **(B**^**v**^**)**. The bar graph shows the relative mean frequency distribution ±SEM of *Actb* intensity in pouches and the soma of microglia from 3 to 4 mice (*N* = 3–4). One-way ANOVA followed by Holm–Šídák’s multiple comparison test. **p < 0.01; n.s, not significant **(B**^**vi**^**)**. **(C)** Graph showing the correlation between the relative levels of IMP1 protein and *Actb* mRNA in 4 (*N* = 4) LPS-injected mice. The asterisk indicates a significant correlation (*p* < 0.05). The data underlying this Figure can be found in S8 Data.

As a first step, we addressed the presence of phagocytic pouches *in vivo* to determine if active ribosomal protein Rsp6, IMP1/ZBP1, and *Actb* mRNA were regulated in these peripheral structures in response to LPS. We focused on the subgranular zone of the dentate gyrus, where naturally occurring cell death associated to neurogenesis can be detected in 1-month-old mice [47]. Indeed, apoptotic cells surrounded by microglial pouches were readily detected in this brain region (p in micrographs and 3D reconstructions shown in Figs 8A, 8B, and S10A). Thus, we quantified levels of pS6 in phagocytic microglia and despite no changes were observed in LPS- compared to saline-injected mice (S10A^i^ and S10A^ii^ Fig), a trend toward an increase of pS6 in pouches compared to the soma was observed in the latter, while this increase was significant in animals that received LPS (S10A^iii^–S10A^vi^ Fig). These results suggest an enrichment of active protein synthesis in phagocytic PeMPs. Importantly, both IMP1/ZBP1 and *Actb* mRNA showed an overlapping pattern in phagocytic microglia (Fig 8A and 8B), being both selectively increased in phagocytic pouches (Fig 8A^i^–8A^v^ and 8B^i^–8B^v^) but not in the soma in response to LPS (see linescans in Fig 8A and 8B). Indeed, both markers were enriched in pouches compared to the soma in LPS-injected animals (Fig 8A^vi^ and 8B^vi^) and a significant positive correlation among them was observed (Fig 8C). These results could indicate that IMP1/ZBP1 drives the localization of *Actb* mRNA to phagocytic PeMPs *in vivo*.

**Fig 9:**
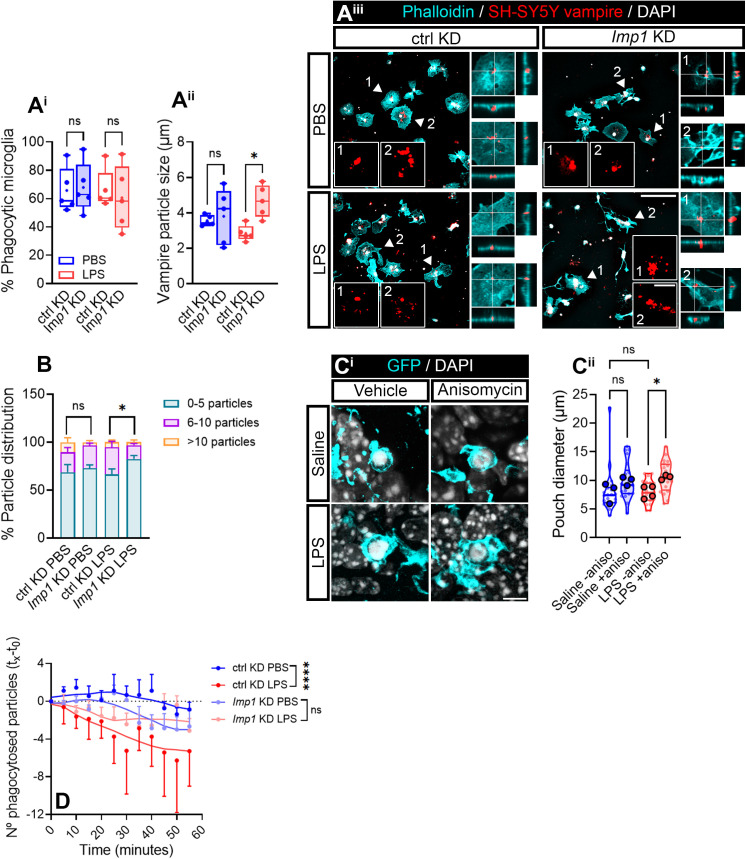
IMP1/ZBP1 downregulation impairs phagocytosed particle degradation. **(A)** Effect of Imp1 KD on LPS-induced microglial phagocytosis. Percentage of control- (ctrl KD) and *Imp1*-transfected (*Imp1* KD) phagocytic microglia after treatment with SH-SY5Y apoptotic neurons labeled with vampire for 1 h after by exposure to PBS or LPS for 30 min in control-transfected and *Imp1* KD cells. Two-way ANOVA followed by Holm–Šídák’s multiple comparison test was performed; n.s, not significant **(A**^**i**^). Vampire particle size following the same experimental setup as before. Two-way ANOVA followed by Holm–Šídák’s multiple comparison test. *p < 0.05; n.s, not significant **(A**^**ii**^). Both graphs (**A**^**ii**^ and **A**^**ii**^**)** correspond to 5 independent experiments (*n* = 5). Representative micrographs of vampire-positive neurons (red) inside phalloidin-stained microglia are shown. Insets depict examples of vampire particles inside phagocytic microglia. Zoomed orthogonal views are also represented for each experimental condition. Scale bar, 100 µm; 10 µm in insets and orthogonal view **(A**^**ii**^). **(B)** Frequency distribution of phagocytic microglia with 1–5, 6–10, and >10 apoptotic neurons inside, following transfection with control (ctrl KD) and *Imp1* (*Imp1* KD) siRNAs and treated with PBS or LPS for 30 min. The graph represents the mean of 5 independent experiments (*n* = 5). Two-way ANOVA followed by Holm–Sidak’s multiple comparison test was performed, *p < 0.05; n.s, not significant. Only the following comparisons are plotted for simplification purposes: PBS vs. LPS in control-transfected cells, PBS vs. LPS in *Imp1*-transfected cells, control- vs. *Imp1*-transfected cells and exposed to PBS, control- vs. *Imp1*-transfected cells and exposed to LPS. **(C)** Pouch diameter in the dentate gyrus of saline- and LPS-injected animals co-administered with vehicle or anisomycin. The representative micrographs show apoptotic cells (condensed DAPI staining) surrounded by phagocytic pouched expressing GFP. Scale bar µm **(C**^**i**^). Violin plots summarizing the diameter of 20–24 pouches (smaller dots) measured in 3–4 animals per experimental conditions (larger dots). One-way ANOVA followed by Holm–Šídák’s multiple comparison test. *p < 0.05; n.s, not significant **(C**^**ii**^). **(D)** Time-course analyses of the number of vampire particles phagocytosed by microglia after washout of excess apoptotic neurons. The graph represents the average particle number ±SEM within 7–9 microglia imaged in 3 independent experiments. Two-way ANOVA followed by Holm–Šídák’s multiple comparison test. ****p < 0.0001; n.s, not significant. The data underlying this Figure can be found in S9 Data.

To determine if IMP1/ZBP1 is actively involved in phagocytosis, we treated cultured microglia with PBS or LPS for 30 min prior to adding apoptotic cells. We fed cells with human SH-SY5Y vampire red transgenic apoptotic neurons for 1 h and analyzed the percentage of microglia with vampire-positive particles inside. Around 60% of untransfected microglia (S10B^i^ Fig) and 70% of control-transfected microglia (Fig 9A^i^) had engulfed apoptotic neurons both in control and LPS-induced conditions. Additionally, *Imp1* KD had no effect on the proportion of phagocytic microglia (Fig 8A^i^). We reasoned that particle engulfment could have reached a plateau after a 1-h exposure to apoptotic neurons, thus we decided to feed cells with for a shorter period. Again, the proportion of phagocytic microglia remained unchanged after exposure to vampire neurons for 10 or 30 min upon LPS treatment in both ctrl and *Imp1* KD cells (S10C Fig). However, microglia fed with apoptotic neurons for 1 h showed smaller vampire engulfed particles in response to LPS in untransfected cells (S10B^ii^ and S10Bi^ii^ Fig) and control-transfected cells (S10B^iv^ Fig). Particle size increased in LPS-treated microglia upon *Imp1* genetic silencing (Fig 9A^ii^ and 9A^iii^) and interestingly, protein synthesis inhibition increased the size of phagocytic pouches in LPS-injected mice (Fig 9C). Moreover, IMP1/ZBP1 downregulation led to a higher proportion of LPS-treated microglia with a smaller number of vampire particles (1–5) and a decrease in cells with higher number of phagocytosed particles (6–10; Fig 9B). These results suggest a role of IMP1-mediated mRNA localization and protein synthesis in efficient particle degradation. To test this possibility, we treated microglia with apoptotic neurons for 1 h and the excess of dead cells was then removed. The number of engulfed particles per microglia was followed over time by live imaging. We observed that the number of SH vampire particle gradually decreased over the span of 55 min in control-transfected microglia exposed to LPS but not to PBS. Interestingly, this effect was blocked in *Imp1* KD cells (Fig 9D). Thus, although LPS does not affect the engulfment step of phagocytosis in microglia, it does seem to enhance degradation in an IMP1-dependent manner.

In summary, our data confirm that acute inflammation drives RNA localization to PeMPs. In addition, we identified *Actb* as one of the transcripts whose localization to and translation in PeMPs is induced by the inflammatory cue LPS. As in other polarized cells, *Actb* localization to microglial PeMPs is regulated by the RBP IMP1/ZBP1, and our results suggest that IMP1/ZBP1-dependent localization of *Actb* and other functionally related transcripts to the periphery of microglia is involved in fundamental microglial functions, including filopodia rearrangements, PeMPs motility and polarized migration, and phagocytosed particle degradation.

## Discussion

Here we report that LPS triggers within minutes the translation in PeMPs of two transcripts involved in cell polarization, cytoskeletal rearrangement and migration, one of them being *Actb*. Genetic inhibition of RBP IMP1/ZBP1 impairs the localization of *Actb* and other related transcripts present in the actin cytoskeleton. IMP1 is also directly or indirectly involved in local β-actin synthesis. Finally, IMP1/ZBP1 downregulation leads to defects in LPS-dependent filopodia rearrangements, PeMP motility and migration, and phagocytosis. Thus, the microglial response to inflammation functionally requires RNA localization within PeMPs.

This work was motivated by evidence that microglia, like many other highly polarized cells, sense local changes in their environment with their peripheral processes which respond independently from their cell soma. Local PeMP responses, including process migration or phagocytosis are required to maintain brain homeostasis, which might be imbalanced by pathological insults like neuroinflammation [14,29]. Thus, microglia rely on efficient signaling initiated at their peripheral processes [14], which we reasoned could be accompanied by changes in the local proteome. Local proteomes can be shaped by proteins transported from the soma or by those synthesized locally in PeMPs. Indeed, in neurons, oligodendrocytes, astrocytes, and radial glia, local protein synthesis is required to maintain local protein homeostasis and for cells to accurately respond to environmental cues, as proteins are produced only when and where they are needed [3,8]. In the case of microglia; however, mRNA localization and local translation had not been described until recently [10]. Given the limited information from microglial cells, we sought to explore this molecular mechanism based on the similarities between axonal growth cones and PeMPs.

PeMPs highly resemble axonal growth cones in terms of their morphology and behavior. Both contain fine processes, termed filopodia, which survey their environment. Upon environmental changes that induce migratory responses, filopodia extend or collapse and drive large processes (lamellipodia) toward chemoattractants or away from chemorepellents. During development, growth cones respond to NGF and netrin-1 by locally translating *Par3*, which leads to axonal outgrowth [36]. Similarly, β-actin is locally produced in response to netrin-1 and enables actin cytoskeleton polymerization and reorganization leading to axon elongation [35]. Hence, we asked if *Actb* and *Par3* mRNAs where also locally translated in PeMPs and to what extent was mRNA localization required for the functional response of microglia to inflammation.

Our results indicate that both transcripts are likely locally translated upon inducing an acute inflammatory state in microglia *in vitro*. However, while local β-actin synthesis was accompanied by *Actb* localization to PeMPs in response to LPS, *Par3* was basally present in the periphery of microglia also in control conditions. These results indicate that *Actb* localization might be a limiting step for β-actin local synthesis in response to LPS. Indeed, reducing *Actb* levels in PeMPs via knockdown of its best-known RBP (IMP1) is sufficient to inhibit its local translation. Interestingly, *Actb* mRNA localization to GC-like PeMPs and to phagocytic pouches was driven by LPS also *in vivo*, partially mirroring our observations in culture. These results point toward the physiological relevance of mRNA localization to PeMPs in response to inflammation. It is possible, that enhanced *Actb* levels in PeMPs are not a direct consequence of the active transport of pre-existing mRNA granules from the soma to the periphery, but rather a result of increased transcription, as suggested by the generalized upregulation of *Actb* (and *Par3*) not only in PeMPs but also in the soma in LPS-injected mice. The extent to which transcription regulation is required to enhance *Actb* mRNA levels in the periphery of microglia in response to acute inflammation requires further investigation.

Neither *Actb* nor *Par3* could be detected in PeMPs when challenging microglia with LPS for 24 h *in vitro*, although local protein synthesis increased overall. These results suggest that translation of a distinct cohort of localized mRNAs might be required for microglia to differentially adapt to acute and chronic insults. This is reminiscent of the axonal response to pathological stimuli. For instance, long exposure of axons to Aβ oligomers, involved in Alzheimer’s disease, drives the localization of the transcription factor *Atf4*, which is not triggered by acute Aβ treatment. Importantly, late axonal synthesis of ATF4 itself depends on an early local production of vimentin [32]. Thus, distinct waves of local translation are required for cells to adapt to the duration of stimuli, and local transcriptomes and translatomes likely need to be dynamically regulated in time to continuously support the accurate response of peripheral processes, including PeMPs, to environmental changes.

Not only the duration but also the nature of stimuli might dynamically change the identity of localized transcripts. For instance, although acute lesions and neurodegenerative cues lead to the axonal localization and translation of common mRNAs, other transcripts are uniquely localized in each specific context. One example is that of *Vim* and *Stat3* mRNAs: whereas *Vim* localizes to axons acutely exposed to Aβ and to lesioned axons, *Stat3* only appears in the later [32,48]. In line with these observations, we ourselves have noticed that whereas LPS drives *Actb* localization to PeMPs, ATP does not, at least *in vitro*. Although the lack of effect on *Actb* in ATP-treated cells might be a matter of dosage and time of treatment, ATP might very well affect transcripts other than *Actb*. This points toward the specificity of local microglial transcriptomes to adapt to distinct challenges. It would be therefore interesting to perform sequencing analyses in PeMPs from microglia exposed to different stimuli at different time points to have a better understanding on how dynamic local transcriptomes are in this cell type.

For local translation to occur, not only mRNAs but also components of the protein synthesis machinery must be transported to the target peripheral compartment. Puromycilation assays and puromycin-RNA colocalization analyses performed *in vitro* already suggest the presence of active ribosomes in PeMPs exposed to LPS *in vitro*. Although we cannot rule out that puromycilated peptides detected in PeMPs are a result of active transport of somatically produced proteins, this is highly unlikely in the case of the β-actin detected by Puro-PLA, as cells were exposed to the antibiotic for only 2 min. Further evidence that supports increased localized translation in response to inflammation is the activation of ribosomal protein Rsp6 in PeMPs not only *in vitro* (following LPS exposure for 30 min and 24 h) but also *in vivo*. Rps6 is a component of the small ribosomal subunit S40 and has been mainly studied for its role in protein synthesis. Rps6 can be activated by the mTOR-S6K/p70 axis, and mTOR is considered a master regulator of local protein synthesis in neurons [49,50]. However, mTOR/S6K/S6 signaling has also been studied in the context of autophagy, cell growth, and survival [51]. Thus, to unambiguously demonstrate the activation of the translation machinery in LPS-treated microglia, ribosomal proteins and translation regulators other than Rps6 should be further analyzed in PeMPs.

mRNA localization requires the formation of ribonucleoprotein complexes (RNPs) in which RBPs play major roles. Following transcription, RBPs bind mRNAs through the recognition of *cis-*localization elements typically present in the 3′ untranslated region (3′UTR) of the transcripts [52]. The resulting RNPs are transported by molecular motors in a translationally repressed state. Once localized to their destination and upon stimulation mRNAs are released from RNPs, associate to the localized protein synthesis machinery and translate into proteins [5]. In order to address the functional relevance of mRNA localization in PeMPs, we downregulated IMP1/ZBP1, which was the first RPB associated to the localization of *Actb* to fibroblast lamellipodia [24]. Since then, IMP1-dependent asymmetric distribution of *Actb* and its involvement in local translation have been described in many cell types, including neurons. Importantly, *Imp1* haploinsufficiency decreases *Actb* mRNA localization to axons and limits the regeneration of peripheral nerves upon injury [26]. Thus, we reasoned that limiting the availability of IMP1/ZBP1 in microglia would affect LPS-dependent *Actb* localization and β-actin synthesis in PeMPs and could uncover a functional relevance of mRNA localization in the microglial response to inflammation. We first analyzed IMP1/ZBP1 levels and observed that positive foci were restricted to the soma in control cells *in vitro* but distributed more peripherally upon LPS treatment, partially mimicking the *Actb* distribution in response to LPS. LPS also led to an increase of this RBP in primary PeMPs in injected mice. This results again partially reproduced *Actb* mRNA pattern in response to systemic inflammation *in vivo*. Importantly, genetic silencing experiments did confirm that IMP1/ZBP1 is required for *Actb* mRNA polarization to and localized translation within PeMPs in response to LPS. *Actb* was; however, not the only transcript affected by *Imp1* knockdown. RNA-Seq analyses revealed the regulation of a cohort of previously described IMP1 targets [42] in LPS-treated microglia, which was blocked in *Imp1* KD cells. Among the RNAs whose regulation was lost upon *Imp1* genetic inhibition, we found several transcripts functionally related to *Actb*, including *Arpc1b*, *Tgfb3*, and *Tubb4a*. All three, together with *Actb*, encode proteins involved in cell motility and cytoskeletal rearrangements.

Given the involvement of IMP1 on the localization of mRNAs encoding cytoskeleton regulators, our next obvious step was to address the consequence of *Imp1* knockdown in PeMP behavior. Interestingly, acute LPS exposure *in vitro* seemed to induce very local changes in PeMPs. For instance, LPS drove filopodia rearrangements along lamellipodia but did not affect lamellar extension, and similar results were obtained *in vivo* in which microglial showed a bushier morphology with filopodia emerging from primary processes. In line with these observations, LPS led to increased PeMP random motility in an IMP1-dependent manner without affecting net lamellar protrusion or cell body migration. On the other hand, whole cell migration was not affected by LPS treatment, which conversely induced PeMP-directed motility when restricting somatic migration. It is precisely in both these contexts that IMP1/ZBP1 seemed to play a relevant role. Finally, ATP did not affect polarized PeMP extension. Although the effect of ATP has been studied in microglia, authors have reported whole cell migration [37], whereas PeMP extension when cell body movement is limited has, to our knowledge, not been described. Overall, these results suggest that PeMP motility and cell body migration might be regulated by different mechanisms which might require the localization of distinct transcripts to PeMPs, some of them being IMP1-independent.

One very relevant function carried out by microglia is phagocytosis, which is essential to restore brain homeostasis by removing cell debris and damaged cells upon inflammation. Phagocytosis is performed by PeMPs *in vivo* and requires rapid morphological changes of processes to form the so-called phagocytic pouches, independently from the cell soma [29]. Importantly, ribosome-bound transcripts involved in phagocytosis are enriched in PeMPs* in* *vivo* and *de novo* protein synthesis is required for the formation of phagocytic pouches *ex vivo* [10]. Consistently, our results show an enrichment of active ribosomal protein Rsp6 compared to the soma in LPS-injected mice suggesting that pouch formation in response to inflammation requires local protein synthesis. More importantly, both *Actb* mRNA and IMP1/ZBP1 were increased in phagocytic pouches *in vivo* and both markers positively correlated in LPS-treated mice. Thus, we reasoned that IMP1-dependent *Actb* localization could be required for phagocytosis. Our results *in vitro* show that IMP1/ZBP1 downregulation does not affect the engulfment step of phagocytosis but suggest the involvement of this RBP in efficient degradation of phagocytosed particles.

In recent years, dysregulated RNA localization and localized translation in neurons have been associated to neurological diseases pointing toward the importance of these molecular mechanisms in supporting axonal, dendritic, and synaptic functions, as reviewed [8]. Importantly, many genetic neurological disorders, such as fragile X syndrome, amyotrophic lateral sclerosis, spinal muscular atrophy, or Hungtinton’s disease are characterized by mutations in RBPs, and defects in RNA localization and/or local translation are common to all of them. IMP1/ZBP1 has been reported to bind several mRNAs encoded by autism spectrum disorder genes [53], and our findings indicate that limiting IMP1/ZBP1 availability disrupts mRNA localization to PeMPs and leads to dysfunctional microglia. Thus, it is tempting to speculate that defects in RBPs not only impair local protein synthesis in neurons, but also in microglia contributing to neurological disorders.

One of the limitations of this study is that mechanistic experiments have been performed *in vitro* using siRNAs as the only genetic tools for local translation inhibition, one of the reasons being the difficulty to genetically manipulate microglia by transfection and transduction both *in vitro* and *in vivo* [54]. Now that microglia are finally in the arena of the local translation field, it will be interesting to develop novel tools to address to what extent defects in RBPs and dysregulated local protein synthesis lead to microglia dysfunction and contribute to the development of brain pathologies in disease models. However, our proof-of-concept work already provides an unprecedented mechanistic insight into local translation regulation in microglia and points toward deficient response to inflammation when local β-actin synthesis in PeMPs is impaired.

## Materials and methods

### Ethics statement

All animal protocols followed the European directive 2010/63/EU and were approved by the UPV/EHU Animal Experimentation Ethics Committee (CEEA protocol numbers M20/2022/415, M20/2022/406, M20/2023/225, and M20/2023/219) and the UPV/EHU Ethics Committee for Research with Biological Agents and GMOs (CEIAB protocol numbers M30/2023/226 and M30/2023/220).

### Animals

For microglial cultures, Sprague Dawley rats were bred in local facilities and brains were obtained from P0-P2 postnatal rats. All *in vivo* experiments were performed in 1-month-old mice expressing the EGFP under the control of the colony-stimulating factor 1 receptor (fms-EGFP) (MacGreen, B6. Cg-Tg (Csf1r-EGFP) 1 Hume/J; Jackson Laboratory stock #018549). The fms-EGFP mouse colony, which expresses GFP at the same physiological levels as CSF1R was established in Achucarro by Dr. Amanda Sierra.

### Primary microglia cultures

Cortical microglial cells were isolated from mixed glial cultures. Briefly, brain hemispheres were dissected from postnatal Sprague Dawley rats (P0-P2) and dissociated with 0.25% trypsin (Sigma Aldrich, Merck, Darmstadt, Germany) and 0.004% DNAse (Sigma Aldrich) for 15 min at 37°C. Trypsinization was stopped by adding glial plating medium containing IMDM (Gibco, Thermo Fisher Scientific, Waltham MA, USA), 10% fetal bovine serum Hyclone (Cytiva, Thermo Fisher Scientific) and 10% of a mixture of antibiotics and antimycotics (Gibco). Cells were homogenized and centrifuged at room temperature for 6 min at 1,200 rpm, resuspended in 1 mL plating medium and mechanically dissociated using 21G and 23G needles. Cells were centrifuged at room temperature for 6 min at 1,200 rpm, resuspended in plating medium and seeded onto 75 cm^2^ flasks (BioLite, Thermo Fisher Scientific). Cultures were maintained at 37°C in a 5% CO^2^ humidified incubator. After 1 day *in vitro* (DIV) the medium was replaced with glucose Dulbecco’s Modified Eagle’s Medium (DMEM, Gibco) containing 10% fetal bovine serum (Sigma Aldrich), 1 U/mL penicillin, 1 µg/mL streptomycin, and 292 µg/mL glutamine (all from Gibco). The medium was replaced every 3 days.

For microglia isolation, 10–17 DIV mixed glial culture flasks were agitated at 180 rpm for 4 h at 37°C. The medium was collected and centrifuged at room temperature for 6 min at 1,200 rpm. Cells were resuspended in serum-free medium containing Neurobasal, 1× B-27 supplement and 2 mM L-glutamine (all from Gibco). Microglia were seeded on Poly-D-lysine (PDL)-coated coverslips (40,000–80,000 cells/cm^2^), on 1- and 3-µm pore transwells (60,000–80,000 cells/cm^2^) or in 35 mm µ-Dish polymer coverslip bottom dishes (Ibidi, Gräfelfing, Germany) (90,000–100,000 cells/cm^2^). Treatments were performed at 12–14 DIV or 19–21 DIV.

### *In vitro* pharmacological treatments

LPS from *Escherichia coli* (LPS, BioXtra, Sigma Aldrich) were added to cells at 150 ng/mL for 30 min or 24 h. Adenosine-5′-O-(3-thio) triphosphate tetralitium (ATP, Tocris Bioscience, Bristol, UK) was added to cells at 300 mM for 30 min. PBS was used as vehicle control.

For puromycilation assays, cells were exposed to 2 µM puromycin dihydrochloride from *Streptomyces alboniger* for 2, 10, or 30 min (Sigma Aldrich). Water was used as a vehicle control. Whenever stated, cells were preincubated for 30 min with anisomycin (Sigma Aldrich) at 40 μM to block protein synthesis.

### *In vivo* pharmacological treatments

One-month-old MacGreen mice (both male and female) were intraperitoneally administrated with LPS (1 mg per kg of body mass in 0.9% saline solution, BioXtra) or control saline solution (NaCl 0.9%) daily for 4 consecutive days, with an interval of 24 h between injections. To address the involvement of protein synthesis in our analyses, saline- and LPS-injected animals received intraperitoneal injections of anisomycin (10 mg per kg of body mass dissolved in 0.9% saline solution) or saline (NaCl 0.9%) at day 3 and 4 after the beginning of the procedures. Twenty-four hours after the last injections, mice were euthanized by anesthesia with ketamine (100 mg per kg of body mass) and xylazine (10 mg per kg of body mass) followed by transcardiac perfusion with NaCl 0.9%. Brains were removed, fixed in cold 4% paraformaldehyde (PFA) overnight at 4°C, dehydrated by sucrose infiltration and kept in OCT or in antifreeze solution until processed. Throughout the entire procedure, mice were monitored for signs of systemic inflammation such as reduced activity/lethargy, hunched posture, or piloerection among others [55] (these symptoms are described in file S9 Data).

### Immunocytochemistry

Microglia were fixed for 20 min at 4°C in 4% PFA, 4% sucrose in PBS. Cells were washed three times for 5 min with PBS, permeabilized and blocked for 30 min in 3% bovine serum albumin (BSA), 100 mM glycine, and 0.25% Triton X-100 (all from Thermo Fisher Scientific). Cells were incubated overnight at 4°C with primary antibodies against puromycin (mouse monoclonal 1:500, Merck), Par3 (rabbit polyclonal 1:1,000, Sigma Aldrich), β-actin (rabbit polyclonal 1:200, Sigma Aldrich), IMP1/ZBP1 (mouse monoclonal 1:500, MBL, Woburn MA, USA or rabbit monoclonal 1:50, Abcam, Cambridge, UK), calreticulin (rabbit polyclonal 1:500, Abcam, Cambridge, UK), pS6 (rabbit monoclonal 1:1,000, Cell signaling technology, Danvers, Massachusetts, USA), Iba1 (guinea pig, polyclonal 1:500, Synaptic Systems, Göttingen, Germany) and P2Y12 (rabbit plyclonal 1:1,000, Sigma Aldrich). Samples were washed three times for 5 min with PBS and incubated with the corresponding fluorescently labeled secondary antibodies for 1 h at room temperature (all from Invitrogen, Thermo Fisher Scientific). Cells were washed three times with PBS and, whenever stated, labeled with Alexa Fluor 488-conjugated phalloidin (1:800, Thermo Fisher Scientific) in PBS or Alexa Fluor 647-conjugated phalloidin (1:1,000, Abcam) in 1% BSA for 30 min at room temperature. Samples were washed three times with PBS and mounted with Prolong Gold Antifade mounting medium with DAPI (Invitrogen, Thermo Fisher Scientific).

### Immunohistochemistry

For immunohistochemistry, we typically obtained 40 µm-thick vibratome sagittal sections from brain samples. Briefly, brains from perfused mice were transferred to a solution containing 30% ethylene glycol and 30% sucrose in deionized water overnight. Sections were obtained in a Leica vibratome (Leica, Wetzlar, Germany) and stored at −20°C until used. Samples were washed three times for 5 min with PBS, permeabilized and blocked for 30 min in 3% BSA, 100 mM glycine, and 0.25% Triton X-100 (all from Thermo Fisher Scientific). Brain sections were then treated with an anti-mouse IgG (donkey polyclonal 1:1,000, Jackson Immunoresearch, West Grove, Pensilvania, USA) for 1 h at room temperature to block unspecific signal and incubated overnight at 4°C with primary antibodies against pS6 (mouse monoclonal 1:1,000, Cell signaling technology), IMP1/ZBP1 (mouse monoclonal 1:500, MBL, Woburn, Massachusetts, USA), GFP (chicken polyclonal 1:1,000, Aveslab, Davis, California, USA) and/or P2Y12 (rabbit polyclonal 1:1,000, Sigma Aldrich) in 3% BSA containing blocking solution. Samples were washed three times for 5 min with PBS and incubated with the corresponding fluorescently labeled secondary antibodies (all from Invitrogen, Thermo Fisher Scientific) together with (DAPI. 4′, 6-Diamino-2-phenylindole, 5 mg/mL, Sigma Aldrich). Sections were washed three times with PBS and mounted with Prolong Gold Antifade mounting medium (Invitrogen, Thermo Fisher Scientific).

### SYTO RNA select labeling

Cultured cells were washed once with cold PBS, once with 50% methanol in PBS and fixed in cold 100% methanol for 5 min. Samples were rehydrated by washing them in 50% methanol in PBS once and in PBS three times at room temperature. Cells were incubated for 20 min at room temperature with 500 nM SYTO RNA Select green fluorescent dye in PBS (Invitrogen). Cells were washed three times with PBS and mounted with Prolong Gold Antifade mounting medium with DAPI (Invitrogen).

### Fluorescent *in situ* hybridization (FISH)

The FISH protocol was performed as previously described [36]. Cells were fixed for 20 min at 4°C in 4% PFA, 4% sucrose in PBS. RNA was precipitated by washing cells with 50%, 75%, and 100% ethanol, and rehydrated by washing once with 50% ethanol, and twice with PBS. Cells were permeabilized and blocked for 30 min in 3% BSA, 100 mM glycine, and 0.25% Triton X-100 (all from Thermo Fisher Scientific). For antigen retrieval, cells were incubated for 10 min with 1 µg/mL proteinase K (Thermo Fisher Scientific) at room temperature, washed three times with PBS, fixed for 10 min with 4% PFA and 4% sucrose in PBS, and washed three times for 5 min with 3% BSA, 100 mM glycine, and 0.25% Triton X-100.

Cells were incubated with 50 ng of *Actb*, *Par3*, or *Gfp* (negative control) targeting riboprobes labeled with digoxigenin in hybridization buffer containing 50% formamide (Sigma), 2× SSC (Sigma), 0.2% BSA, 1 mg/mL *E.coli* tRNA (Sigma), 1 mg/mL salmon sperm DNA (Fisher Scientific) for 3 h and 30 min at 37°C. As a negative control, GFP targeting probes were used. Cells were washed with 50% formamide in 2× SSC once for 30 min at 37°C, once with 50% formamide in 1× SSC for 30 min at 37°C, three times with 1× SSC for 15 min each, and three times with tris-buffered saline (TBS) solution containing 0.1% Tween at room temperature for 5 min each.

Samples were blocked and permeabilized for 30 min in 3% BSA, 0.1% Tween in TBS at room temperature. Cells were incubated overnight at 4°C with primary antibodies against digoxigenin (mouse monoclonal 1:500, Sigma), β-actin (rabbit polyclonal 1:200, Sigma), and/or Iba1 (guinea pig, polyclonal 1:500, Synaptic Systems, Göttingen, Germany) in 3% BSA, 0.1% Tween in TBS. Cells were washed three times with TBS containing 0.1% Tween at room temperature for 5 min each and incubated with the appropriate fluorophore-conjugated secondary antibodies for 1 h at room temperature. Samples were washed with PBS twice for 5 min. Coverslips were air-dried and mounted with Prolong Gold Antifade mounting medium with DAPI (Invitrogen, Thermo Fisher).

All the digoxigenin-conjugated riboprobes were synthetized with the T7 promoter (…GCCCTATAGTGAGTCGTATTAC-3′) which were preceded by the following specific mRNA targeting sequences at the 3′ end:

*Actb.1*: 5′-AACCGTGAAAAGATGACCCAGATCATGTTTGAGACCTTCAACACCCCAGC−3′.
*Actb.2*: 5′-ATGTGGATCAGCAAGCAGGAGTACGATGAGTCCGGCCCCTCCATCGTGCA−3′.
*Actb.3*: 5′-AGACCTCTATGCCAACACAGTGCTGTCTGGTGGCACCACCATGTACCCAG-3′.
*Actb.4*: 5′-GAGCGTGGCTACAGCTTCACCACCACAGCTGAGAGGGAAATCGTGCGTGA-3′.
*Actb.5*: 5′-TCCCTGGAGAAGAGCTATGAGCTGCCTGACGGTCAGGTCATCACTATCGG-3′.
*Par3.1*: 5′-GCCGTGCGGAGATGGCCGCATGAAAGTTTTCAGCCTTATCCAGCAGGCGG-3′.
*Par3.2*: 5′-TTCCACGAGAATGACTGCATTGTGAGGATTAACGATGGAGATCTTCGAAA-3′.
*Par3.3*: 5′-GGACGGTGGGATTCTAGACCTGGATGACATCCTCTGTGACGTTGCCGATG-3′.
*Par3.4*: 5′-TGCTTTTCGGCCTTATCAAACCACAAGTGAAATTGAGGTCACGCCTTCAG-3′.
*Par3.5:* 5′-TGCAGATTTGGGGATCTTCGTTAAGTCCATCATTAACGGGGGAGCTGCAT-3′.
*Egfp.1:* 5′-GATGCCACCTACGGCAAGCTGACCCTGAAGTTCATCTGCACCACCGGCAAG-3′.
*Egfp.2*: 5′-GACCACATGAAGCAGCACGACTTCTTCAAGTCCGCCATGCCCGAAGGCTAG-3′.
*Egfp.3*: 5′-ACTTCAAGGAGGACGGCAACATCCTGGGGCACAAGCTGGAGTACAACTACG-3′.
*Egfp.4*: 5′-AAGCAGAAGAACGGCATCAAGGTGAACTTCAAGATCCGCCACAACATCGAG-3′
*Egfp.5*: 5′-AGTTCGTGACCGCCGCCGGGATCACTCTCGGCATGGACGAGCTGTACAAGG-3′.

A similar FISH protocol was followed in mouse brain sections (cortical samples), except that 33.5 ng/μL of *Actb* or *Par3* targeting riboprobes were used in this case. After anesthesia with 100 mg/kg ketamine and 10 mg/kg xylazine, 1-month-old mice (male and female) were transcardially perfused with NaCl 0.9%. Brains were removed and fixed in cold 4% PFA in PBS overnight at 4°C. Samples were dehydrated first in 15% sucrose in PB 0.1M and later in 30% sucrose in PB 0.1M, cryopreserved in OCT (Thermo Fisher Scientific), and 10 µm-think serial sagittal sections were obtained in a cryostat (Leica, Wetzlar, Germany). Sections were stored at −80°C until used. Brain samples were co-stained with antibodies against GFP (chicken polyclonal 1:1,000, Aveslab) and P2Y12 (rabbit polyclonal 1:1,000, Sigma Aldrich) in this case, and counterstained with DAPI.

In the case of *Actb* detection in hippocampal microglia, we used vibratome-processed sections, given that 10 µm-thick samples were not appropriate to visualize phagocytic pouches.

### Puromycilation coupled with proximity ligation assays (PuroPLA)

Cells were treated with 250 ng/mL LPS (Sigma) or vehicle as previously described and exposed to 2 μM puromycin (Sigma) for 2–10 min. Samples were fixed in 4% PFA and 4% sucrose in PBS, washed three times with PBS for 5 min, and permeabilized and blocked for 30 min in 3% BSA, 100 mM glycine, and 0.25% Triton X-100 (all from Thermo Fisher Scientific). Cells were incubated overnight at 4°C with an anti-puromycin antibody (mouse monoclonal 1:500, Merck) combined with an anti-β-actin antibody (rabbit polyclonal 1:1,000, 07-Sigma) or an anti-Par3 antibody (rabbit polyclonal 1:200, Sigma). Detection of newly synthesized β-actin or Par3 through PuroPLA was performed according to the manufacturer’s instructions, using rabbit PLAplus and mouse PLAminus probes and red Duolink detection reagents (Sigma Aldrich). Before mounting with Duolink in situ mounting medium with DAPI (Sigma Aldrich), coverslips were incubated with Alexa Fluor488-conjugated phalloidin (1:800; Thermo Fisher Scientific) for 30 min at room temperature.

### Western blotting

Proteins were extracted from cultured microglia using RIPA buffer supplemented with 1× protease and phosphatase inhibitors (Thermo Fisher Scientific) and separated by PAGE-SDS electrophoresis in 4%–12% Tris-Glycine polyacrylamide continuous gradient gels in reducing conditions. Proteins were transferred onto polyvinylidene difluoride membrane (Amersham, Sigma-Aldrich) previously activated with methanol. Membranes were blocked in 5% nonfat dried milk in TBS-T and incubated overnight at 4°C with IMP1/ZBP1 antibody (mouse monoclonal 1:500 dilution in 5% nonfat dried milk in TBS-T, MBL). After 3 washes with TBS-T, membranes were incubated with the appropriate HRP-conjugated secondary antibody for 1 h at room temperature. Detection was performed with the SuperSignal West Pico PLUS kit (Thermo Fisher Scientific) and imaged with the ChemiDoc MP system (Bio-Rad, Hercules, CA, USA). Total protein was visualized with Ponceau S staining solution (Thermo Fisher Scientific) and with Pierce Reversible Protein Stain Kit (Thermo Fisher Scientific).

### *Imp1* knockdown experiments

*Imp1* genetic silencing was performed using siRNAs in serum-free medium in the absence of antibiotics. Double-stranded siRNAs were transfected with lipofectamine RNA iMAX (Invitrogen, Thermo Fisher) following manufacturer recommendations. Cells were transfected for 6 or 24 h with Imp1-targeting siRNAs (siRNA #1: s155193, or siRNA #2: s155193. Both were from Ambion, Thermo Fisher Scientific).

The sequences of *Imp1*-targeting siRNAs are the following:

siRNA #1: 5′-GGAAAAUACAGAUCCGGAAtt – 3′
siRNA #2: 5′ – GCCUGAGAAUGAGUGGGAAtt-3′

Transfection efficiency was analyzed by immunocytochemistry with an anti-IMP1/ZBP1 antibody (mouse monoclonal 1:500, MBL).

### RNA sequencing

Microglia were seeded on top of a 1 µm-pore membrane of a transwell system. Cells were transfected with a control siRNA or an *Imp1*-targeting siRNA, and the lower compartment was then treated with vehicle or LPS. After removing the cell material from the upper compartment with a cotton swab (see S6A Fig for details), total RNA from PeMPs was isolated using the Direct-zol RNA Miniprep kit following manufacturer instructions (ZYMO Research, Irvine, California, USA). RNA quality was addressed in a Bioanalyzer electrophoresis system (Agilent Technologies, Santa Clara, California, USA). After removing rRNAs, 2 ng of RNA was used to prepare total RNA libraries with the SMARTer Stranded Total RNA-Seq Kit (v3, Takara Bio, Kusatsu, Japan). Sequencing was performed in a NovaSeq 6000 instrument (paired-end, 2 × 100 bp. Illumina, San Diego, California, USA). Reads were aligned to the rat refence genome (mRatBN7.2) using HISAT2 [39] and differential localization to PeMPs was analyzed with DESeq2 [40] (both resources can be found at https://usegalaxy.org/). Clustering of RNAs of interest was performed with Metascape [41] and STRING [43]. Venn diagrams represented in figures were based on results retrieved from InteractiVenn [56]. Whenever possible, functional clusters identified by STRING were named after the most represented pathway identified by the WikiPathways database [57] (FDR < 0.05). If WikiPathways did not yield any hit, we used Reactome Pathway [58] or KEGG Pathway [59] databases instead.

### RT-qPCRs

RNA extracts were collected with Trizol from previously desomatized microglial PeMPs and total RNA was isolated using the NZY Total RNA isolation kit (MB13402, Nzytech Lisbon, Portugal), following the manufacturer instructions. The RNA concentration of each sample was determined using a NanoDrop spectrophotometer (Thermo Fisher Scientific). To obtain complementary DNA (cDNA) from the RNAs, whenever possible 100 ng or the totality of RNA samples were subjected to reverse transcription using the SuperScript IV First-Strand Synthesis System (Invitrogen) with oligo(dT)20 and random hexamers as primers following the manufacturer instructions. Due to the low quantity of RNA an additional preamplification step was performed using SsoAdvanced PreAmp Supermix (1725160, Bio-Rad Laboratories, Hercules, California, USA) including primers of all the genes of interest for the reaction and following manufacturer instructions. Once pre-amplified cDNA was obtained, real-time PCR was performed using the Power SYBR Green Master Mix kit (Thermo Fisher Scientific). The reaction conditions were as follows: 40 cycles at 95°C for 10 min, 95°C for 15 s, and 60°C for 1 min.

The sequences of the forward (F) and reverse (R) primers used to pre-amplify and amplify the cDNAs of interest are detailed below (Invitrogen; Sigma-Aldrich). All of the primers were designed based on the Rattus Norvegicus genome. The mean centering method was used to normalize transcript levels.

Actb (Rattus norvegicus):
  F: 5′-TACAACCTTCTTGCAGCTCC-3′
  R: 5′-ATACCCACCATCACACCCTG-3′
Arpc1b (Rattus norvegicus):
  F: 5′-AGGAGAATGACTGGTGGGTG-3′
  R: 5′-CGTTCCTCCACCTCCTTGAT-3′
Bcl6 (Rattus norvegicus):
  F: 5′-AAAGGCCGGACACCAGTTTT-3′
  R: 5′-GGAGGCGGTTAAGGTTGAGA-3′
Dysf (Rattus norvegicus):
  F: 5′-GATGACTTTCTGGGCTCCCT-3′
  R: 5′-CAAGGCCACCATCCTTTCAC-3′
Hnrnpa2b1 (Rattus norvegicus):
  F: 5′-CCTTTGGAGAGGAAAAAGAGAG-3′
  R: 5′-GCTTTCCCCATTGCTCGTAG-3′
Kif1a (Rattus norvegicus):
  F: 5′-CTGGAGGACCCAAATTGACC-3′
  R: 5′-CCTGGAGCAAACAAGATGCG-3′
Kif26a (Rattus norvegicus):
  F: 5′-AATGTCTGGAGGTCGTAGCC-3′
  R: 5′-GTGAGTGTGTGGTCCCTGTA-3′
Map1b (Rattus norvegicus):
  F: 5′-AGTTCTACTTGCTGGTGGTGG-3′
  R: 5′-GAGGATCTTTTGTCCTGGAACT-3′
Tgfb3 (Rattus norvegicus):
  F: 5′-ATGACCCACGTCCCCTATCA-3′
  R: 5′-CTGCCAGTTCATTGTGCTCC-3′
Tubb4a (Rattus norvegicus):
  F: 5′-ACTGGGACCTATCATGGGGA-3′
  R: 5′-GCTCCAGATTGACCAAACACA-3′

### Transwell migration assays

Microglia were obtained from a mixed glial culture as previously described. Cells (60,000–80,000 cells/cm^2^) were seeded in a transwell system which consists of a culture insert with a polyethylene terephthalate membrane that creates two compartments. Cells were seeded onto PDL-coated membranes with 3 µm-pores which enabled microglial migration toward the lower compartment, or with 1 μm-pores in which cell body migration was restricted. The lower compartment was treated with 150 ng/mL LPS, 300 mM ATP or PBS for 30 min. Migration of the nuclei and the cytoskeleton were addressed by DAPI and phalloidin staining respectively.

### *In vitro* phagocytosis assays

The protocol for *in vitro* phagocytosis was adapted from Beccari and colleagues [60]. Briefly, SH-SY5Y cell lines stably transfected with the red fluorophore tFP602 (SH-SY5Y vampire. InnoProt, Derio, Spain) were cultured in DMEM (Gibco, Thermo Fisher Scientific) containing 10% fetal bovine serum (Sigma Aldrich), 10% penicillin/streptomycin/glutamine (Gibco, Thermo Fisher Scientific), and 250 μg/mL geneticin (Gibco, Thermo Fisher Scientific). Apoptosis was induced by adding 3 µM staurosporine (Sigma Aldrich) for 4 h at 37°C. Culture medium was collected and centrifuged at 1,300 rpm for 4 min. Apoptotic cells were washed in warm PBS for 5 min and then centrifuged twice at the same speed and duration. The resulting cell pellet was resuspended in microglia plating medium and added to primary microglial cultures at a 1:3 ratio (SH-SY5Y to microglia) for 10 min, 30 min, or 1 h before fixation, or 1 h for live-cell imaging and subsequent analysis. Prior to adding apoptotic cells, primary microglia were pre-treated with either LPS or a vehicle control for 30 min. Phagocytosis was addressed by quantifying the percentage of phalloidin-labeled microglia containing vampire particles as well as the particle size.

### Live cell imaging

Phagocytic particle degradation was assessed after the removal of nonphagocytosed apoptotic cells by medium replacement 1-h post-incubation. The number and fluorescence of apoptotic particles within three randomly selected microglial cells per condition was quantified starting 45 min after the medium change, over a 50-min period, acquiring images every 5 min.

To address PeMP motility, microglia obtained from a mixed glial culture were seeded in 35 mm µ-Dish polymer coverslip bottom dishes coated with PDL (90,000–100,000 cells/cm^2^. Ibidi). Cells were treated with 150 ng/mL LPS or PBS just before live imaging was performed. Three random fields per sample were imaged for 2 min with 5-s intervals. PeMP motility was addressed in cells immediately treated with vehicle or LPS and after 5, 10, 15, 20, and 30 min of treatment. PeMP motility was measured as the absolute movement of lamellae from a starting position (specified at *t* = 0) regardless of their protrusion or retraction. Average protrusion in cells treated for 10 min is also specified in the Results section.

### Image acquisition and fluorescence quantification

Images were acquired in cultured microglia using an EC Plan-Neofluar 63×/1,40 Oil DIC or an EC Plan-Apochromat 20x/0.8 Air M27 objective on an Axio-Observer Z1 microscope equipped with AxioCam MRm Rev. 3 (Zeiss, Oberkochen, Germany) and Hamamatsu EM-CCD ImagEM (Hamamatsu Photonics, Hamamatsu, Japan) digital cameras. Settings for image acquisition were determined in a random field of a control sample ensuring pixel intensities were within the linear range and avoiding pixel saturation. Images were acquired with ZEN 2 (blue edition) version 2.0.0.0. software (Zeiss). Cells were selected based on the counterstain (e.g., phalloidin) for the blind acquisition of the labeling of interest and settings were kept identical for all sampled cells in any given experiment. Whenever possible, five random fields per coverslip and two coverslips per experimental condition were imaged.

In the case of microglia sampled from cryostat-processed mouse brains, images were acquired using the same EC Plan-Neofluar 20× objective microscope in cryostat slices. GFP-positive cells were randomly imaged from the IV layer of the cortex of 10 µm-thick brain sagittal sections. 6 stacks were imaged per field using structured illumination with Apotome2 (Zeiss) and the summed mean pixel intensity was used for quantification. 6 stacks were imaged per field using structured illumination with Apotome2 (Zeiss) and the summed mean pixel intensity was used for quantification.

Images from vibratome slices were obtained in a confocal microscope (Leica Stellaris 5, DM6 B CS. Leica). GFP-positive cells were randomly imaged from layer IV of the cortex of from the dentate gyrus. Individual GFP+ microglia were imaged with a 40× objective, with bidirectional scanning and optical sections of 0.7 μm z-step interval.

In any case, after background subtraction, the average pixel intensity was calculated for each sample. For presentation in figures, the grayscale images were converted to RGB and contrast and background settings were set the same in control and experimental conditions for the staining of interest. Markers used as counterstain for PeMP selection were adjusted for an optimal visualization in figures.

SYTO-, FISH-, and IMP1/ZBP1-positive foci were measured on binarized images as previously described [31]. The number of objects was scored for each staining in MPPs or the soma with the Analyze Particle function in FIJI/ImageJ (NIH) and normalized to area or PeMP length. Given the discrete numbers of Puro-PLA puncta these were manually quantified on raw images.

### Statistical analysis

The sample size is specified in the figure legends. Statistical analyses were performed with Prism 8 and 10 (GraphPad Software, San Diego, CA, United States) following a randomized block design where samples from the same experiment were matched to eliminate inter-experimental variability, unless matching could not be performed or was statistically not significant. When comparing the means of two groups taking one variable into account, two-tailed *t*-tests were performed. One-way ANOVA followed by Holm–Šidak’s multiple comparison test was used if one variable was compared between more than two groups, unless otherwise stated. If more than one variable was analyzed, we performed two-way ANOVA followed by Holm–Sidak’s or Tukey’s multiple comparison tests.

## Supporting information

S1 Fig Characterization of multiple parameters in primary microglial cultures. **(A)** Depiction of a microglia cell with multiple PeMPs. The inset shows a magnification of a PeMP mainly composed of a lamella from which fine processed (filopodia) emerge. Figure was created in BioRender. Baleriola, J. (2025) https://BioRender.com/28mogjk. **(B)** Expression of microglia markers in primary cultures. Micrographs show primary cultures stained for phalloidin and Iba-1 **(B**^**i**^) or for Iba-1 and P2Y12 **(B**^**ii**^). Scale bar 50 µm. The graphs represent the proportion of phalloidin-positive cells expressing Iba-1 (left) and the percentage of P2Y12-positive cells among the Iba-1-positive population. Labeling was performed in cells from 3 independent cultures (*n* = 3) **(B**^**iii**^). **(C)** To visualize newly synthesized proteins in PeMPs, cells were incubated with vehicle (−puro; 1), with puromycin for 10 and 30 min (+ puro; 2 and 3, respectively) or with anisomycin for 30 min and puromycin for 10 (+ puro 10′ + aniso; 4) or 30 min (+ puro 30′ + aniso; 5). Representative micrographs of puromycin distribution in whole cells and in lamellae are shown. Scale bar, 20 µm (insets, 5 µm) **(C**^**i**^). The graph represents the distribution of puromycin-labeled newly synthesized proteins along lamellae **(C**^**ii**^). **(D)** RNA localization to the periphery of microglia was visualized with SYTO RNASelect green fluorescent dye. To determine if SYTO selectively labeled RNA, fixed cells were treated with DNAse or with RNAse. The box and whisker graph indicates the mean fluorescence intensity of SYTO in phalloidin-labeled microglia from 6 independent experiments (*n* = 6) analyzed by one-way ANOVA followed by Dunnet’s multiple comparison test. **p < 0.01; n.s, not significant **(D**^**i**^). Micrographs show phalloidin and SYTO labeling in control, DNAse- and RNAse-treated cells. Scale bar, 10 µm **(D**^**ii**^). **(E)** Protein synthesis measured in PeMPs following a 30-min LPS exposure. The box and whisker graph indicates the mean fluorescence intensity of puromycin in the periphery of microglia in 6 independent cultures (*n* = 6) analyzed by two-tailed *t* test; n.s, not significant **(E**^**i**^). Th box and whisker graph indicates the mean fluorescence intensity of pS6 in the periphery of microglia in 6 independent cultures (*n* = 6) analyzed by two-tailed *t* test; n.s, not significant. Note that values from PBS-treated cells are the same as in Fig 1Dii as control cells were the same for 30-min and 24-h treatments with LPS. Figures have been separated for simplification purposes **(E**^**ii**^). The data underlying this Figure can be found in S10 Data.
(TIF)

S2 Fig Morphology of microglia in the McGreen mouse brain. **(A)** Representative micrographs of cortical layers IV and V showing the morphological transition from ramified microglia in saline-injected mice to bushy or hyper-ramified in LPS-injected animals. Note that all GFP-labeled microglia are also positive for P2Y12. Scale bar, 50 µm. **(B)** Schematic representation of primary PeMPs, GC-like PeMPs, and phagocytic pouches in hippocampal microglia of 1-month-old mice. The micrographs indicate the nucleus (n in left panel) of a P2Y12-labeled microglia and a nearby apoptotic body engulfed by a phagocytic pouch (in the right panel). Each peripheral structure analyzed *in vivo* are further described in the cartoon on the right. Both scale bars are 5 µm long. Figure was created in BioRender. Baleriola, J. (2025) https://BioRender.com/28mogjk.
(TIF)

S3 Fig *Actb* and *Par3* mRNA levels in microglia upon LPS exposure. **(A)** A prerequisite for localized translation is the presence of mRNAs in cell peripheral structures. We addressed the localization of *Actb* and *Par3* mRNAs to microglial lamella by fluorescent in situ hybridization (FISH) and did not observe any positive signal compared to a negative when cells were exposed to PBS or LPS for 24 h. Representative images of FISH are shown. Scale bar, 20 µm. **(B)** Both *Actb* and *Par3* were readily detected in cells treated with vehicle or LPS for 30 min. Representative images are shown. Scale bar, 20 µm. Asterisks in (A) and (B) indicate the lamellae shown in insets. **(C)** Binarized FISH signals in cells counterstained for β-actin. (1) and (2) in **(C**^**i**^) indicate the PeMPs and the perinuclear region represented in insets in **(C**^**iv**^). Scale bars 20 µm in (C^i^) and 10 µm in (C^iv^). These are the same cells shown in Figs 2B (for *Actb*) and S3B (for *Par3*) without counterstain. The box and whisker plots indicate the number of *Actb* (upper graph) and *Par3* (lower graph) mRNA foci relative to the negative probe quantified in 6 independent experiments (*n* = 6) following a 30-min exposure to PBS and LPS. One-way ANOVA followed by Dunnet’s multiple comparison test. *p < 0.05; **p < 0.01; n.s, not significant **(C**^**ii**^). The box and whisker plots indicate the number of *Actb* (upper graph) and *Par3* (lower graph) mRNA foci relative to the negative probe quantified in 7 independent experiments (*n* = 7) following a 24-h exposure to PBS and LPS. One-way ANOVA followed by Dunnet’s multiple comparison test. n.s, not significant **(C**^**iii**^). The data underlying this Figure can be found in S11 Data.
(TIF)

S4 Fig *Par3* mRNA in PeMPs *in vivo.* **(A)** *Par3* mRNA FISH in cortical microglia (positive for P2Y12 and GFP) from fms-EGFP 1-month-old mice injected with saline or LPS. (1) and (2) indicate GC-like PeMPs and cell bodies represented in insets. Scale bars 10 µm (5 µm insets). **(B)** Linescans summarize the mean distribution ±SEM of the FISH signal from the nucleus to the PeMPs in 49–72 individual cells per condition. **(C)** Violin plots represent the mean intensity of *Actb* in 25–36 sampled GC-like PeMPs (smaller dots) and 49–72 cell bodies (smaller dots) from 3 to 4 mice (larger dots). Two-tailed *t* tests. **p < 0.01; ****p < 0.0001. **(D)** The box and whisker graph shows the mean relative frequency distribution ±SEM of *Par3* intensity in PeMPs and the soma in cortical microglia from 3 to 4 mice (*N* = 3–4). One-way ANOVA followed by Holm–Šídák’s multiple comparison test. ***p < 0.001; ****p < 0.0001. The data underlying this Figure can be found in S12 Data.
(TIF)

S5 Fig ATP does not alter *Actb* mRNA levels and LPS specifically affects this transcript in Iba-1 cells. **(A)** *Actb* levels in microglia in response to ATP. Linescans represent the distribution of the FISH signal from the nucleus to the PeMPs in 43−55 individual cells per condition **(A**^**i**^). Estimation plots show *Actb* levels in ATP-treated cells relative to controls in PeMPs and the soma measured in 6−7 independent experiments (*n* = 6−7). Two-tailed *t* tests. n.s, not significant **(A**^**ii**^). **(B)** Relative levels of *Actb*-positive foci in binarized images. Representative micrographs of *Actb* distribution (binarized images) in PBS- and ATP-treated cells expressing Iba-1. (1) and (2) indicate the PeMPs and the perinuclear regions, respectively, represented in insets. Scale bars, 20 µm (5 µm insets) **(B**^**i**^). The box and whisker graph shows the relative frequency distribution of *Actb* foci in the periphery and the soma of microglia after treatment with PBS, ATP, or LPS for 30 min in 6−7 independent experiments (*n* = 6−7). One-way ANOVA followed by Holm–Šídák’s multiple comparison test. *p < 0.05; **p < 0.01; ***p < 0.001; n.s, not significant **(B**^**ii**^). **(C)** Actb mRNA distribution in Iba-1-negative cells in response to LPS. Linescans represent the distribution of *Actb* FISH signal from the nucleus to the PeMPs in 21 individual nonmicroglial cells per condition **(C**^**i**^). Estimation plots show *Actb* levels in LPS-treated cells both negative **(C**^**ii**^) and positive **(C**^**iii**^) for the microglia/macrophage marker Iba-1. Graphs show the relative amounts of the transcript measured in 6 independent experiments (*n* = 6). *p < 0.05; n.s, not significant. The data underlying this Figure can be found in S13 Data.
(TIF)

S6 Fig LPS enhances localized *Actb* and *Par3* translation in the periphery of microglia. Relative levels of RBP IMP1/ZBP1 in microglia in untransfected and transfected cells. Effect of *Imp1* KD on *Actb.* **(A)** New synthesis of β-actin in microglial peripheral structures was assessed with a 10-min puromycin pulse in PBS- and LPS-treated cells followed by proximity ligation assay (PLA) with antibodies against puromycin and β-actin. As a negative control, cells were preincubated with the protein synthesis inhibitor anisomycin. The box and whisker graph represents the average PLA puncta within lamellae in phalloidin-stained microglia treated with PBS or LPS in 6 independent cultures (*n* = 6) and analyzed by pairwise comparisons with Holm–Šídák’s correction. **p < 0.01; n.s, not significant **(A**^**i**^). Representative micrographs of PLA puncta in LPS-treated cells are shown. Scale bar, 10 µm (insets, 5 µm) **(A**^**ii**^). **(B)** New synthesis of Par3 in PeMPs was assessed with a 10-min puromycin pulse in PBS- and LPS-treated cells followed by PLA with antibodies against puromycin and Par3. As a negative control, cells were preincubated with the protein synthesis inhibitor anisomycin. The box and whisker graph represents the average of PLA puncta within lamellae in phalloidin-stained microglia treated with PBS or LPS in 6 independent cultures (*n* = 6) and analyzed by pairwise comparisons with Holm–Šídák’s correction. *p < 0.05; n.s, not significant **(B**^**i**^). Representative micrographs of PLA puncta in LPS-treated cells are shown. Scale bar, 10 µm (insets, 5 µm) **(B**^**ii**^). **(C)** IMP1/ZBP1 levels in PBS- and LPS-treated microglia. Representative micrographs show IMP1 immunostaining in phalloidin-positive cells treated with vehicle or LPS. Scale bar 20 µm **(C**^**i**^). The box and whisker graph represents the relative IMP1 levels in microglia exposed to PBS or LPS for 30 min in 4 independent experiments (*n* = 4). Two-tailed *t* test. n.s, not significant **(C**^**ii**^). **(D)** IMP1/ZBP1 levels in PBS- and LPS-treated microglia. Representative western blot showing IMP1/ZBP1 and total protein levels following exposure to PBS or LPS for 30 min **(D**^**i**^). The bar graph summarizes the quantification of western blots obtained from 3 independent cultures (*n* = 3). Two-tailed *t* test; n.s, not significant **(D**^**ii**^). **(E)** Knockdown efficiency of two nonoverlapping siRNAs targeting *Imp1* compared to a negative siRNA following 6 and 24 h of transfection. The box and whisker graph represents the mean intensity of IMP1 protein in cells transfected with a control siRNAs and two *Imp1* siRNAs from 5 to 6 independent experiments (*n* = 5–6). Two-tailed *t* tests was performed, *p < 0.05; **p < 0.01; n.s, not significant, siRNA #1 versus ctrl siRNA (dashed line) and siRNA #2 versus ctrl siRNA (dashed line) **(E**^**i**^). Representative micrographs of transfected cells are shown. Scale bar, 20 µm **(E**^**ii**^). **(F)** *Imp1* knockdown (KD) alters *Actb* mRNA localization toward the periphery of microglia. To test the specificity of the FISH probes, *Actb* foci were normalized to the signal from nontargeting probes (dashed lines). The box and whisker graph represents the average *Actb*-positive foci (relative to the negative control) detected within lamellae in microglia treated with PBS or LPS and transfected with a control (ctrl KD) or an *Imp1*-targeting (Imp1 KD) siRNA in 5 independent experiments (*n* = 5). One-way ANOVA followed by Holm–Šídák’s multiple comparison test. *p < 0.05; n.s, not significant **(F**^**i**^). Representative micrographs of *Actb* distribution (binarized images) counterstained with β-actin protein. These cells are the same as those shown in Fig 4B without counterstain. Scale bars, 20 µm **(F**^**ii**^). The data underlying this Figure can be found in S14 Data.
(TIF)

S7 Fig Effect of IMP1/ZBP1 downregulation in transcripts localized to PeMPs in response to acute inflammation localized analyzed by RNA-Seq. **(A)** Functional annotation, using Metascape, of significantly changed transcripts (LPS vs. PBS) in control **(A**^**i**^) and *Imp1* siRNA-transfected cells **(A**^**ii**^). Only the top 20 categories from all significantly changed terms (*p* < 0.05) are represented in the figure. **(B)** Functional interaction between *Actb* and transcripts whose LPS-induced regulation in PeMPs is blocked by *Imp1* knockdown (red nodes). Blue and green nodes indicate the functional clustering of other RNA not associated with Actb. Confidence = 0.4; FDR = 0.05; MCL clustering = 1.4. The data underlying this Figure can be found in S15 Data.
(TIF)

S8 Fig Effect of IMP1/ZBP1 in PeMP morphology and motility in response to acute inflammation. **(A)** Lamellar morphology in PBS- and LPS-treated microglia lamellae length **(A**^**i**^) and width **(A**^**ii**^) were analyzed in 3 independent experiments (*n* = 3) as shown in box and whisker plots. Two-tailed *t* tests. n.s, not significant. **(B)** Lamellar morphology in PBS- and LPS-treated microglia transfected with a control or an *Imp1*-targeting siRNA. Lamellae length **(B**^**i**^) and width **(B**^**ii**^) were analyzed 6 independent experiments (*n* = 6) as shown in box and whisker plots. One-way ANOVA followed by Holm–Šídák’s multiple comparison test. n.s, not significant. **(C)** PeMP motility analyzed by life cell imaging in untransfected cells treated with vehicle or LPS 10 min. Cell visualization was performed for 2 min (5-s cycles). Representative micrographs of lamellar behavior every 30 s are shown **(C**^**i**^). The box and whisker graph represents the net protrusion of lamellae after a 10-min treatment in cells cultured in 5 independent experiments (*n* = 5). One-way ANOVA followed by Holm–Šídák’s multiple comparison test. n.s, not significant **(C**^**ii**^). **(D)** PeMP motility analyzed by life cell imaging in ctrl KD and *Imp1* KD cells treated with vehicle or LPS 10 min. Cell visualization was performed for 2 min (5-s cycles). Representative micrographs of lamellar behavior every 30 s are shown **(D**^**i**^). The box and whisker graph represents the net protrusion of lamellae after a 10-min treatment in cells cultured in 4 independent experiments (*n* = 4). One-way ANOVA followed by Holm–Šídák’s multiple comparison test. n.s, not significant. **(D**^**ii**^). The data underlying this Figure can be found in S16 Data.
(TIF)

S9 Fig Effect of acute LPS exposure on lamellar polarized migration. **(A)** PeMP migration in a 1 µm-pore transwell culture system. Somatic migration was restricted in this culture setup, and only peripheral processes were observed in the lower chamber. We treated the lower compartment and assessed polarized PeMP migration by the area covered by phalloidin in PBS- and LPS-treated cells. Box and whisker graph represents F-actin-rich cytoskeleton extension (phalloidin staining) measured in 6 independent experiments (*n* = 6). Two-tailed *t* test was performed, *p < 0.05; n.s, not significant **(A**^**i**^). Micrographs depicting nuclear migration (DAPI) and F-actin extension (phalloidin) toward the lower compartment are shown. Scale bar, 100 µm **(A**^**ii**^). LPS effect on cytoskeleton extension was addressed in control- (ctrl) transfected cells to determine the reproducibility of our results compared to those in untransfected cells. Despite the variability, we did observe a consistent relative effect on polarized PeMP migration in LPS- compared to PBS-treated cells. Two-tailed *t* test was performed. *p < 0.05 **(A**^**iii**^). **(B)** To address microglial migration, cells were cultured in 3-µm-diameter transwell membranes. The percentage of cells migrated to the lower side of the membrane **(B**^**i**^), as well as the relative coverage of phalloidin staining **(B**^**ii**^) were calculated in 5 independent experiments (*n* = 5) and are represented in the box and whisker graphs. One-way ANOVA followed by Holm–Šídák’s multiple comparison test. n.s, not significant. PBS or LPS were applied to the bottom of the transwell for 30 min. Representative images of cells migrated to the lower side of the membrane and those remaining in the upper compartment are shown. Scale bar, 50 µm **(B**^**iii**^). **(C)** Microglial cell body migration was also addressed in transfected cells in 3 µm-pore membranes. Representative micrographs of nuclear (DAPI) and F-actin (phalloidin) staining in the lower and upper compartments of transwells is shown. Scale bar, 100 µm **(C**^**i**^). Again, cell migration was analyzed as the percentage of microglial nuclei (DAPI) found in the lower side of the chamber with respect to the total nuclei (DAPI) found in both the upper and lower chambers **(C**^**ii**^), and the covered by phalloidin in the lower compartment was also measured **(C**^**iii**^). Both box and whisker plots represent the results from 5 independent experiments (*n* = 5). Two-tailed *t* test were performed, n.s, not significant (C^ii^ and C^iii^). **(D)** PeMP migration in a 1 µm-pore transwell culture system in response to ATP was addressed in 1 µm-pore transwells. Representative images of lamellae (phalloidin- and Iba-1-positive) migrated to the lower side of the membrane in response to ATP and LPS are shown. Scale bar, 50 µm **(D**^**i**^). The box and whisker graph represents the relative coverage of phalloidin staining at the lower side of the membrane analyzed in 5 independent cultures (*n* = 5). One-way ANOVA followed by Dunnet’s multiple comparison test. *p < 0.05; n.s, not significant **(D**^**ii**^). The data underlying this Figure can be found in S17 Data.
(TIF)

S10 Fig pS6 levels in PeMPs *in vivo* and effect of IMP1/ZBP1 on phagocytosis. **(A)** Levels of phosphorylated ribosomal protein Rsp6 in hippocampal phagocytic microglia (positive for P2Y12 and GFP) from fms-EGFP 1-month-old mice injected with saline or LPS. Linescans represent the distribution of the pS6 from the nucleus to the PeMPs in 9–11 individual cells per condition **(A**^**i**^). Violin plots represent the mean intensity of pS6 in 23–24 sampled pouches (smaller dots) and 9–1 cell bodies (smaller dots) from 3 to 4 mice (larger dots). Two-tailed t tests. n.s, not significant **(A**^**ii**^). Representative micrographs show pS6 immunostaining in the cell body (s) and in phagocytic PeMPs (p) of microglia from saline- and LPS-injected mice. A series of 5 consecutive optical sections (i–v) are shown. Scale bar 10 µm **(A**^**iii**^). 3D reconstructions of microglial cells shown in **(A**^**iii**^) and stained for IMP1 pS6 in saline- **(A**^**iv**^) and LPS-injected **(A**^**v**^) mice. Images might have been rotated to position phagocytic pouches in the foreground. Scale bar 5 µm. The box and whisker graph shows the relative frequency distribution pS6 intensity in phagocytic PeMPs and the soma in hippocampal microglia from 3 to 4 mice (*N* = 3–4). One-way ANOVA followed by Holm–Šídák’s multiple comparison test. *p < 0.05; n.s, not significant **(A**^**vi**^). **(B)** Percentage of phagocytic microglia and size of phagocytosed particles in response to LPS. Cultured microglia pretreated with PBS or LPS for 30 min were fed for 1 h with human SH-SY5Y apoptotic neurons labeled with vampire. The percentage of microglia with vampire-positive particles inside was quantified (+Vamp) compared to a negative control (−Vamp). Box and whisker graphs from 7 independent experiments (*n* = 7) show no differences in phagocytic microglia in PBS or LPS treatments **(B**^**i**^). However, particles were smaller in LPS-treated cells compared to controls as represented from the box and whisker plots from 7 independent experiments (*n* = 7). Two-tailed *t* test was performed, **p < 0.01 **(B**^**ii**^). Vampire particle size in PBS- and LPS-treated microglia is shown in representative micrographs. Scale bar 50 µm **(B**^**iii**^). LPS effect on vampire particle size was addressed in control- (ctrl) transfected cells to determine the reproducibility of our results compared to those in untransfected cells. Despite the variability, we did observe a consistent relative effect on the size of vampire particles in phagocytic microglia in LPS- compared to PBS-treated cells. Two-tailed *t* test was performed, *p < 0.05, **(B**^**iv**^). **(C^i^)** Percentage of phagocytic microglia after 10- and 30-min exposures to apoptotic neurons. Cultured microglia exposed with PBS or LPS for 30 min and treated with human SH-SY5Y apoptotic neurons labeled with vampire. The box and whisker graphs show no differences between experimental conditions. 6 independent experiments were performed at each time point. The data underlying this Figure can be found in S18 Data.
(TIF)

S1 Raw Images Uncropped membranes related to S6Di and 5Ai Figs. The two upper membranes show raw western blot images of lanes represented in S6Di Fig. The two lower membranes shown raw western blot images of lanes represented in Fig 5Ai. Samples selected for image presentation are indicated, as well as the molecular weight (KDa) marker.
(PDF)

S1 Data Raw data related to Fig 1. Contains 5 spreadsheets with raw data corresponding to Fig 1Bii, 1Ci, 1Cii, 1Dii, 1Eii, and 1Eiii as indicated.
(XLSX)

S2 Data Raw data related to Fig 2. Contains 6 spreadsheets with raw data corresponding to Fig 2Ai, 2Aii, 2Av, 2Bi, 2Bii, and 2Bv as indicated.
(XLSX)

S3 Data Raw data related to Fig 3. Contains 4 spreadsheets with raw data corresponding to Fig 3Ai, 3Aii, 3Aiv, and 3Bii as indicated.
(XLSX)

S4 Data Raw data related to Fig 4. Contains 6 spreadsheets with raw data corresponding to Fig 4Ai, 4Aii, 4Aiv, 4Bi, 4Biii, and 4Biv as indicated.
(XLSX)

S5 Data Raw data related to Fig 5. Contains 6 spreadsheets with raw data corresponding to Fig 5Ai, 5Bi, 5Bii, 5Cii, and 5Dii as indicated.
(XLSX)

S6 Data Raw data related to Fig 6. Contains 6 spreadsheets with raw data corresponding to Fig 6Bi, 6Bii, 6C, 6D, 6E, and 6F as indicated.
(XLSX)

S7 Data Raw data related to Fig 7. Contains 7 spreadsheets with raw data corresponding to Fig 7Aii, 7Aiii, 7Aiv, 7Bii, 7Biii, 7Ci, 7Ciii, and 7Dii as indicated.
(XLSX)

S8 Data Raw data related to Fig 8. Contains 7 spreadsheets with raw data corresponding to Fig 8Aii, 8Av, 8Avi, 8Bii, 8Bv, 8Bvi, and 8C as indicated.
(XLSX)

S9 Data Raw data related to Fig 9. Contains 5 spreadsheets with raw data corresponding to Fig 9Ai, 9Aii, 9B, 9Cii, and 9D as indicated.
(XLSX)

S10 Data Raw data related to S1 Fig. Contains 5 spreadsheets with raw data corresponding to S1Biv, S1Ciii, S1Di, S1Ei, and S1E^ii^ Fig as indicated.
(XLSX)

S11 Data Raw data related to S3 Fig. Contains 2 spreadsheets with raw data corresponding to S3Ci1 and S3Civ Fig indicated.
(XLSX)

S12 Data Raw data related to S4 Fig. Contains 3 spreadsheets with raw data corresponding to S4B, S4C, and S4D Fig as indicated.
(XLSX)

S13 Data Raw data related to S5 Fig. Contains 6 spreadsheets with raw data corresponding to S5Ai, S5Aii, S5Bii, S5Ci, S5Cii, and S5Ci^ii^ Fig as indicated.
(XLSX)

S14 Data Raw data related to S6 Fig. Contains 6 spreadsheets with raw data corresponding to S6Ai, S6Bi, S6Cii, S6Dii, S6Ei, and S6F^ii^ Fig as indicated.
(XLSX)

S15 Data Raw data related to S7 Fig. Contains 2 spreadsheets with raw data corresponding to S7Ai, S7Aii, and S7B Fig as indicated.
(XLSX)

S16 Data Raw data related to S8 Fig. Contains 6 spreadsheets with raw data corresponding to S8Ai, S8Aii, S8Bi, S8Bii, S8Ci, and S8Di Fig as indicated.
(XLSX)

S17 Data Raw data related to S9 Fig. Contains 5 spreadsheets with raw data corresponding to S9Ai, S9Aii, S9Aiii, S8Bi, S8Bii, S8Cii, S8Ciii, and S8D^ii^ Fig as indicated.
(XLSX)

S18 Data Raw data related to S10 Fig. Contains 7 spreadsheets with raw data corresponding to S10Ai, S10Aii, S10Avi, S10Bi, S10Bii, S10Biv, and S10Ci Fig as indicated.
(XLSX)

S19 Data Inflammation symptoms in LPS-injected mice. The spreadsheet reports external signs of inflammation in mice injected with saline or LPS for four consecutive days. Mice received co-injections of saline or anisomycin at days 3 and 4 of the procedure together with saline or LPS.
(XLSX)

## Abbreviations

ATP: adenosine triphosphate
BSA: bovine serum albumin
cDNA: complementary DNA
DMEM: Dulbecco’s Modified Eagle’s Medium
DIV: day *in vitro*
EGFP: enhanced green fluorescent protein
FISH: fluorescent *in situ* hybridization
GO: gene ontology
IMDM: Iscove's modified Dulbecco's medium
IMP1/ZBP1: IGF2 mRNA binding protein 1/zipcode binding protein 1
LPS: lipopolysaccharides
PBS: phosphate-buffered saline
PDL: Poly-D-lysine
PeMPs: peripheral microglial processes
PFA: paraformaldehyde
PLA: proximity ligation assay
RBP: RNA-binding protein
RNPs: ribonucleoprotein complexes
RT-qPCR: reverse-transcription quantitative polymerase chain reaction
siRNAs: small-interference RNAs
TBS: tris-buffered saline
