# Supplementary material for: RNA-binding protein IMP1/ZBP1 directs local translation in microglial processes to regulate motility and phagocytosis during inflammation
Source: PLoS Biol. 2025 Nov 10;23(11):e3003463. doi: 10.1371/journal.pbio.3003463 (PMC12599960; doi:10.1371/journal.pbio.3003463)

IMP1/ZBP1 (related to S6D^{i} Fig)

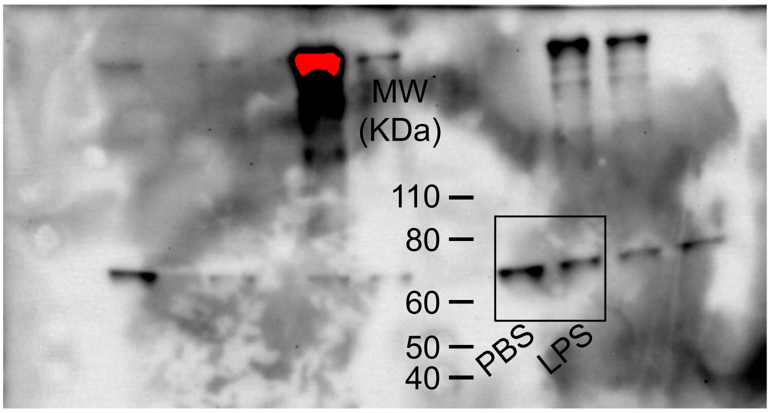

Total protein stain (related to S6D^{i} Fig)

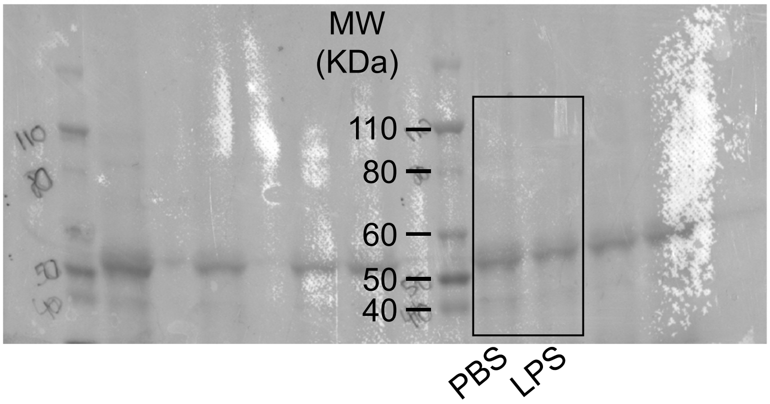

IMP1/ZBP1 (related to Fig 5A^{i})

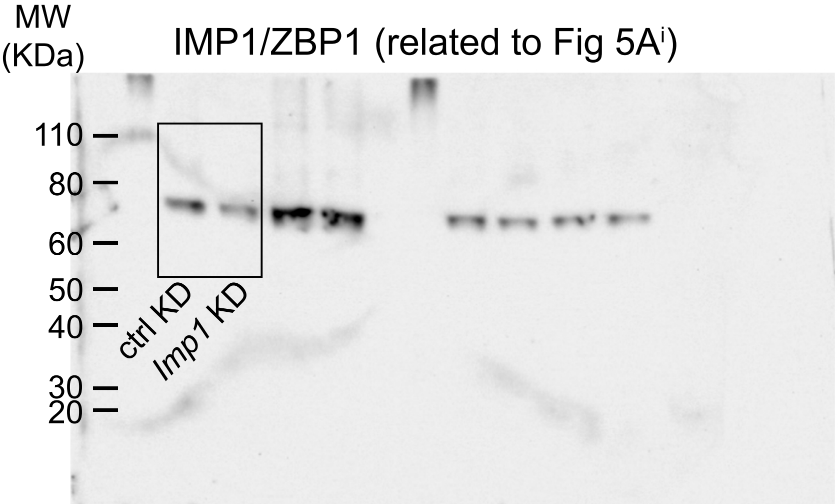

Total protein stain (related to Fig 5A^{i})

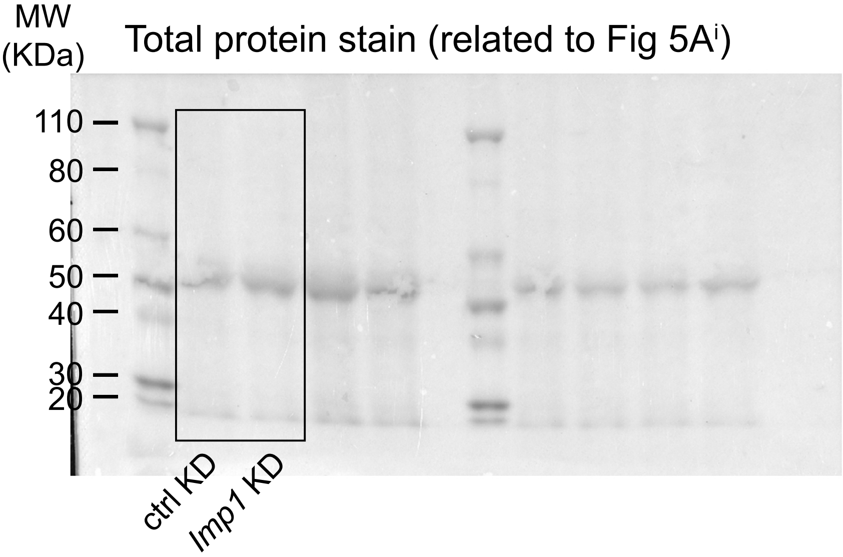

Supplement: S1 Raw Images — The two upper membranes show raw western blot images of lanes represented in S6Di Fig. The two lower membranes shown raw western blot images of lanes represented in Fig 5Ai. Samples selected for image presentation are indicated, as well as the molecular weight (KDa) marker. (PDF) [file pbio.3003463.s011.pdf]
